# Modulating Macrophage Polarization for Severe Acute Pancreatitis Therapy via Cisplatin-like Prussian Blue Nanozymes

**DOI:** 10.7150/thno.113523

**Published:** 2025-08-11

**Authors:** Ling Wu, Rui Cai, Yuhang Li, Shuqi Liao, Yinghui Song, Yufeng Li, Jishan Li, Donghong Yu, Zhong Cao, Sulai Liu

**Affiliations:** 1 Hunan Provincial Key Laboratory of Materials Protection for Electric Power and Transportation & Hunan Provincial Key Laboratory of Cytochemistry, School of Chemistry and Pharmaceutical Engineering, Changsha University of Science and Technology, Changsha 410114, China.; 2 Department of Hepatobiliary Surgery/Central Laboratory, Hunan Provincial People's Hospital (The First Affiliated Hospital of Hunan Normal University), Changsha 410005, China.; 3 Hunan Engineering Research Center of Digital Hepatobiliary Medicine, Changsha 410005, China.; 4 Hunan Provincial Key Laboratory of Biliary Disease Prevention and Treatment, Changsha 410005, China.; 5 State Key Laboratory of Chemo/Bio-Sensing and Chemometrics, College of Chemistry and Chemical Engineering, Hunan University, Changsha 410082, China.; 6 Department of Chemistry and Bioscience, Aalborg University, Aalborg, DK-9220, Denmark.

**Keywords:** Severe acute pancreatitis, cisplatin-like Prussian blue nanozyme, high biosafety, ROS scavenger, macrophage polarization

## Abstract

**Rationale:** Development of reactive oxygen species (ROS) antioxidants with high biosafety and anti-inflammatory properties for macrophage regulation in severe acute pancreatitis (SAP) therapy remains challenging. Here, we engineered a cisplatin-like calcium hexacyanoferrate Prussian blue nanozyme (Cri-Pt-CaFe_PB_) that functioned as a ROS scavenger to modulate macrophage polarization.

**Methods:** The Cri-Pt-CaFe_PB_ was prepared using a stepwise freeze-thaw method, and its structure and long-term stability were characterized by transmission electron microscope and dynamic light scatting. Subsequently,* in vitro* experiments were conducted to investigate the cytotoxicity and protective effects of Cri-Pt-CaFe_PB_. Fluorescence imaging and ICP-MS were applied to monitor its biodistribution and pharmacokinetics* in vivo*. Moreover, the biochemical kits, immunofluorescence, hematoxylin-eosin staining, and western blot were utilized to clarify *in vivo* therapeutic effect of Cri-Pt-CaFe_PB_ in SAP mice.

**Results:** Pt(VI) precursor was covalently coordinated with ultramicro CaFe_PB_ nanospheres (~5 nm) and then converted into Pt(II) cisplatin-like nanozyme-based antioxidants, exhibiting exceptional ROS scavenging and anti-inflammatory effects at cellular and molecular levels with no toxicity *in vitro* or *in vivo*. Density functional theory simulation reveals the key role of Cri-Pt-CaFe_PB_ with high peroxidase activity to the anti-inflammation treatment. Remarkably, the Cri-Pt-CaFe_PB_ can protect the pancreas from oxidative stress damage and induce M1 to M2 macrophages repolarization after intravenous administration by downregulating CD86 protein expression (an M1 marker) and activating Arg-1 protein (an M2 marker), effectively reversing inflammatory damage in SAP and inhibiting the expression of proinflammatory cytokines.

**Conclusions:** This study highlights the feasibility of Cri-Pt-CaFe_PB_ nanozymes as ROS scavengers to regulate macrophage polarization towards M2 phenotype, which offers a novel effective nanomedicine strategy.

## Introduction

Severe acute pancreatitis (SAP) is a gastrointestinal emergency frequently associated with organ failure lasting more than 48 h and carries a high fatality rate 1-3. High infiltration of pancreatic macrophages is a key event in the early stages of SAP 4. In particular, macrophages can be functionally classified into proinflammatory M1- or anti-inflammatory M2-polarizing phenotypes, each playing distinct roles in SAP 5, 6. For example, M1 macrophages primarily polarize at the site of inflammation in the initial stage, where they release proinflammatory factors and reactive oxygen species (ROS), intensifying inflammation 7. Meanwhile, M2 macrophages can secrete anti-inflammatory cytokines, which contribute to tissue repair and structural reconstruction 8. An imbalanced ratio of M1/M2 macrophages is strongly associated with SAP progression and severity 9. Importantly, ROS are a hallmark pathological feature of SAP, stimulating and regulating macrophages differentiation towards proinflammatory M1 subtype, thus accelerating the progression of SAP 10, 11. Moreover, excessive ROS production can also destabilize the cellular microenvironment and cause irreversible damage to biological macromolecules such as DNA, nucleic acids, and proteins, all of which are considered drivers of SAP development 2. Therefore, modulating macrophage polarization while mitigating oxidative stress constitutes an effective strategy for SAP treatment.

Recently, artificial nanozymes with highly tunable multienzyme-like activities and biocompatibility 12-15, including cerium, vanadium, metal, iron oxide, metal-organic frameworks, Prussian blue (PB), and single-atom nanozymes have been widely used to treat inflammation disorder by ROS scavenging 16-19 . Among these, Prussian blue analogs (PBAs) are a unique class of nano-catalytic materials that substitute iron (Fe_4_^3+^[Fe^2+^(CN)_6_]_3_) with other metals, such as cobalt, nickel, zinc, copper, and manganese, etc. 20, 21. Approved by the U.S. Food and Drug Administration for the treatment of poisoning caused by thallium and other radioactive elements, PBA based nanomaterials exhibit excellent stability, customizable functional surfaces, and multienzyme-like activities, such as peroxidase (POD), superoxide dismutase (SOD), and glutathione peroxidase (GPx). These properties make PBA-based nanozymes highly effective in treating various inflammatory diseases. Xie *et al.* proposed a monodisperse Prussian blue nanozyme with inherent antioxidant and anti-inflammatory bioactivities, which can improve acute pancreatitis by inhibiting TLRs/NF-κB signaling pathway 22. Yao *et al.* reported a novel therapeutic agent comprising curcumin-modified cobalt-doped Prussian blue analogs 23. In a mildly acidic tissue environment, the release of curcumin and the valence state changes of Co(III) and Fe(II) aid in macrophage regulation and ROS clearance, which can effectively treat ulcerative colitis. Zhang *et al.* designed a biomimetic simvastatin-loaded porous manganese substituted Prussian blue (PMPB) that can mitigate ROS and inflammation in atherosclerosis, while releasing Mn^2+^ from PMPB to enhanced MRI 24. Acid-responsive hollow mesoporous Prussian blue nanoparticles were engineered for the synergistic treatment of acute pancreatitis through calcium ion homeostasis regulation and inhibition of acinar cell autodigestion 25. Zhang *et al.* utilized the excellent drug loading potential of Mn-modified mesoporous Prussian blue to load cibotium barometz, as a nanoplatform to alleviate intervertebral disc herniation by inhibiting oxidative stress and inflammation 26. Additionally, Wang *et al.* designed ultrasmall KCa(H_2_O)_2_[Fe^III^(CN)_6_]·H_2_O Prussian blue nanomaterial with multienzyme activity, which is known as a robust ferroptosis inhibitor and can effectively treat acute kidney injury via scavenging endogenous reactive oxygen/nitrogen species (RO/NSs) 27. Despite significant progress in removing ROS from PBA and its doping modifications, there are still challenges in balancing the M1/M2 macrophage ratio and elucidating the relationship between nanozymes structure and therapeutic efficacy at the cellular and molecular levels.

**Scheme 1:**
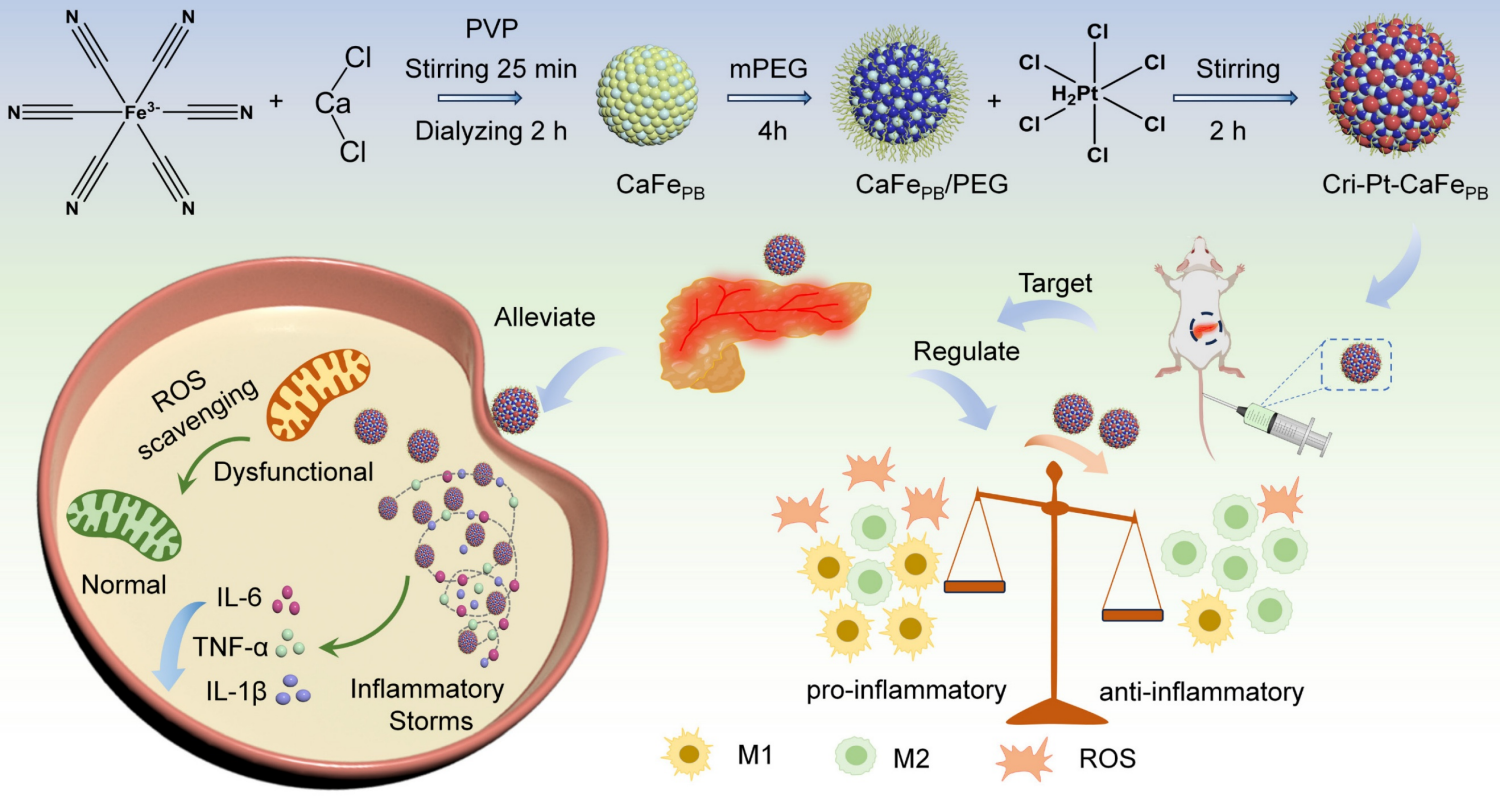
Schematic diagram for synthesis pathway of Cri-Pt-CaFe_PB_ and their anti-inflammatory effects through ROS scavenging and macrophage polarization modulation in SAP.

It is interesting that Pt(IV) compounds are easily reduced to FAD-approved cisplatin(II) drugs and provide more coordination sites for further modification, making them suitable to serve as linkers for grafting onto PBA 28, 29, which can greatly improve the antioxidant properties of the nanozyme materials and reduce their biological toxicity during treatment. In this study, a precursor of Pt(IV) was covalently coupled to a PEG-coated ultrastructural calcium hexacyanoferrate Prussian blue analog (CaFe_PB_) using a stepwise freeze-thaw method. The Pt(IV) precursors were then converted to cisplatin- and Pt(IV)-conjugated calcium hexacyanoferrate Prussian blue analogs (Cri-Pt-CaFe_PB_) to alleviate SAP. It is worth noting that the use of cisplatin-like nanozyme not only improves their biosafety but also enhances their enzyme-like activity. As illustrated in Scheme 1, the as-synthesized Cri-Pt-CaFe_PB_ exhibits excellent antioxidant and anti-inflammatory effects through ROS clearance, protecting cells from oxidative damage and maintaining normal metabolism. Density functional theory (DFT) simulations have further revealed the therapeutic mechanism of these nanozymes, proving that the POD-like activity of Cri-Pt-CaFe_PB_ plays a decisive role in its anti-inflammatory effects. In the mouse model of SAP, treatment with Cri-Pt-CaFe_PB_ can restore the pancreas to near-normal tissue structure. Importantly, we also find that Cri-Pt-CaFe_PB_ can regulate macrophage polarization, while clearing ROS and promoting the transformation of M1 macrophages into M2's. Moreover, it can effectively downregulate the levels of proinflammatory cytokines *in vivo* and inhibit the death of pancreatic cells in SAP. Thus, our study provides a feasible strategy for the use of cisplatin-like Prussian blue nanozyme to treat SAP through the modulation of macrophage polarization and ROS scavenging, which broadens the biological application of nanozymes in the treatment of inflammation or ROS-related diseases.

## Methods

### Preparation of Cri-Pt-CaFe_PB_

A cisplatin-like calcium hexacyanoferrate Prussian blue nanozyme (Cri-Pt-CaFe_PB_) was prepared via a stepwise freeze-thaw method. Firstly, a homogeneous solution was prepared by mixing CaCl_2_ (20 mL, 6 mM), K_3_[Fe(CN)_6_ (20 mL,10 mM), and 500 mg of polyvinylpyrrolidone (PVP), and stirred at 25 °C for 20 min. Subsequently, the mixed solution was transferred to a dialysis bag with molecular weight cut-off (MWCO) of ~12,000 and dialyzed for 3 h, with a distilled water for replacement every 30 min. The obtained solution was then frozen at -20 °C for 12 h. After thawing naturally, 40 mL of nanozyme aqueous dispersion was poured into 10 mg of Mal-mPEG_2000_-COOH and stirred uniformly at a constant speed under darkness for 4 h. The PEG-coated calcium hexacyanoferrate Prussian blue analog (CaFe_PB_) block solid products were obtained by drying in an oven at 60 °C. The obtained bulk solid CaFe_PB_ was subsequently re-dissolved in distilled water (40 mL). Finally, the above CaFe_PB_ solution was mixed with H_2_PtCl_6_∙6H_2_O (1 mg/mL) solution in a volume ratio of 9:1 with stirring for 2 h and then dried in an oven at 60 °C, yielding Cri-Pt-CaFe_PB_ nanozyme.

### Preparation of CaFe-Pt_PB_

A Pt-doped calcium hexacyanoferrate Prussian blue analog (CaFe-Pt_PB_) was synthesized by a one-step freeze-thaw method. In brief, a homogeneous solution was prepared by mixing 20 mL of CaCl_2_ (6 mM), 20 mL of K_3_[Fe(CN)_6_(10 mM), and 500 mg of PVP. Then this solution was mixed with 1.0 mg/mL H_2_PtCl_6_∙6H_2_O in a volume ratio of 9:1, followed by stirring at 25 °C for 25 min. The obtained solution was transferred to a dialysis bag (MWCO ~12,000). After dialysis for 3 h, the solution was frozen at -20 °C for 12 h to yield CaFe-Pt_PB_. The obtained nanozyme was further modified by adding Mal-mPEG_2000_-COOH (10 mg), stirring for 4 h in darkness and drying in an oven at 60 °C to obtain CaFe-Pt_PB_. In addition, platinum calcium (PtCa_PB_) and platinum iron (PtFe_PB_) Prussian blue nanomaterials were synthesized following the procedures of CaFe-Pt_PB_, in which water was used instead of potassium ferricyanide.

### POD-like activity

The POD activity of nanomaterials was evaluated through monitoring the characteristic absorption peak of oxidized 3,3',5,5'-tetramethylbenzidine (TMB) at 652 nm. Specifically, 100 μL of 100 mM hydrogen peroxide (H_2_O_2_) solution with 40 mM TMB and 100 μL of 200 µg/mL nanozymes were added to 800 μL of PBS (0.01 M pH=4.0) solution in a colorimetric dish. After reacting for 30 s, the absorbance of the colorimetric system was recorded at 652 nm to evaluate POD activity of nanozymes.

### •OH scavenging

The hydroxyl radical (•OH) scavenging activity was examined by both the salicylic acid method and electron paramagnetic resonance spectroscopy (EPR). For former, a reaction system (1000 μL) was obtained by mixing ferrous sulfate (FeSO_4_, 7.5 mM, 85 μL), H_2_O_2_ (0.1 mM, 85 μL), sampling materials (200 µg/mL, 20 μL), and 810 μL of distilled water. After incubation at 37 °C for 30 min, the •OH scavenging ability of nanozymes was analyzed by measuring the change in the UV-vis absorbance of 2,3-dihydroxybenzoic acid (DHBA) at 510 nm. In addition, the •OH scavenging ability of the nanozymes was also determined via EPR spectroscopy. 5,5-Dimethyl-1-pyrroline N-oxide (DMPO) is applied to trap •OH produced by Fenton reagent (FeSO_4_/H_2_O_2_). In detail, a reaction mixture containing DMPO (100 mM, 50 μL), H_2_O_2_ (50 mM, 20 μL), 20 μL of 0.5 mM FeSO_4_, and 50 μL of 0.2 mg/mL nanozyme was added to 20 mM pH=3.5 NaAc-HAc buffer (500 μL of the system). The reaction system without nanozymes was used as a blank control. The •OH removing capability of each nanozyme was assessed by EPR amplitude intensity.

### Assays of SOD-like activity

The SOD-like activity of the nanozymes was estimated by investigating the inhibition of pyrogallol (PG) autoxidation 30. Briefly, 50 mM, pH=8.2 Tris-HCl buffer (1.4 mL) and PG solution (0.015 M, 50 μL) were mixed with 50 μL of distilled water. After reacting for 3 min, 50 μL of nanozyme solution (0.2 mg/mL) was vigorously added to the above solution by rapid shaking. The absorbance at 325 nm (A/min) was measured every 30 s for 4 min. The reference solution was a reaction mixture without nanozymes.

### Scavenging of RNS

The scavenging activity of the nanozymes against 1,1-diphenyl-2-trinitrophenylhydrazine radical (DPPH•) free radicals was measured using anhydrous ethanol as a solvent. Each nanozyme with the same concentration (200 µg/mL, 20 μL) was mixed with a dispersion of DPPH (200 μg/mL, 160 μL) anhydrous ethanol. After incubation at 37 °C in the dark for 30 min, the absorbance at 517 nm was measured to assess the DPPH• scavenging ability of the different nanozymes.

### Scavenging of ONOO•

The solutions of NaNO_2_ (50 mM, 10 mL) and H_2_O_2_ (25 mM, 10 mL) were mixed to generate ONOO• with stirring for 3 min. Upon the addition of HCl (1 M, 5 mL) and NaOH (1.5 M, 5 mL) under stirring, the reaction mixture turned pale yellow within 1 s. Then, 200 μL of each nanozyme solution (200 μg/mL) was mixed with 600 μL of the yellow solution for 30 s. The whole reaction system was maintained in an ice-water bath. After incubation for 1 min, the absorbance at 302 nm was recorded to measure the ONOO- scavenging ability of the nanozyme.

### GPx-like ability

The GPx ability of the nanozymes was measured by oxidizing glutathione (GSH) to oxidized glutathione (GSSG) in the presence of H_2_O_2_. Specifically, 100 μL of 10 mg/mL GSH, 33 μL of 10 mg/mL reducing coenzyme (NADPH), 62 μL of 20 units/mL glutathione reductase (GR), and 50 μL of 200 µg/mL nanozyme were added to a 730 μL of 0.01 M PBS buffer solution (pH = 7.4) containing 25 μL of 0.01 M H_2_O_2_ solution. Finally, the absorbance of the reaction solution at 340 nm was recorded as a function of time.

### Live-dead cell experiment

A fluorescent dye was prepared by adding 20 μL of Calcein-AM/propidium iodide (AM/PI) solution with a fixed volume ratio of 3:1 together with 5 mL of 1X assay buffer. Cells were cultured in T-25 flasks at 37 °C and 5% CO_2_ in a constant-temperature incubator, that details are described in Supporting Information. After successful implantation of the cells, inflammatory responses were induced by the addition of 10 μg/mL lipopolysaccharide (LPS) for 24 h. The experimental samples were divided into three groups: normal control group (0.9% NaCl-treated), SAP group (LPS-treated, 10 μg/mL), and nanozyme treatment group (Cri-Pt-CaFe_PB_, 200 μg/mL). After incubation with Cri-Pt-CaFe_PB_ nanozyme for 24 h, the cells were washed three times with PBS. Subsequently, the prepared fluorescent dye was incubated with the cells for 15 min, followed by three times washing with PBS buffer. Finally, fluorescence images of the cells were captured using a Leica DMi8 microscope.

### Intracellular ROS detection

To determine the production of intracellular ROS, 2',7'-Dichlorodihydrofluorescein diacetate (DCFH-DA) staining was employed. Under different oxidative damage conditions, the cells were divided into three groups: a control group treated with 0.9% NaCl, an SAP group treated with LPS (10 μg/mL), and an experimental group pretreated with LPS (10 μg/mL) followed by incubation with the Cri-Pt-CaFe_PB_ nanozyme for 24 h. Following these treatments, the cells were incubated with 10 μM of DCFH-DA for 15 min and washed three times with Dulbecco's modified Eagle's medium (DMEM) to remove excess dye. Subsequently, the treated cells were photographed using a Leica DMi8 microscope. For flow cytometry analysis, the cells were further treated with DCFH-DA (10 μM) for 15 min, followed by digestion with trypsin to prepare a single-cell suspension. Intracellular ROS levels were measured using a FACSCanto II flow cytometer.

### Cell proliferation analysis

The cells were seeded in six-well plates and divided into different subgroups on the basis of the experimental design. The SAP and experimental groups were exposed to a LPS solution (10 μg/mL in PBS) for 24 h to induce the pathological characteristic of SAP 31. Then, the experimental group continued to be incubated with the Cri-Pt-CaFe_PB_ nanozyme for 24 h, whereas the SAP (LPS-treated) and control (0.9% NaCI) were treated with the same amounts of DMEM medium. The pretreated cells were subsequently treated with 5-ethynyl-2'-deoxyuridine (EdU, 10 μM) in a constant-temperature incubator (37 °C) for 2 h. Subsequently, the cells were washed three times with PBS to remove unbound EdU and other impurities. Then, the cells were co-cultured with AlexaFluor-488 antibody under dark condition, and counterstained with DAPI for nuclear staining to facilitate subsequent fluorescence observation. Finally, the EdU-labeled DNA was carefully observed and recorded via a Leica DMi8 microscope.

### Biosafety assessment

All mice (ICR, female, 6-week-old) were purchased from Henan Skobes Biotechnology Co., Ltd. All operations on the mice in this study were approved by the Institutional Animal Committee of Hunan Provincial People's Hospital (The First Affiliated Hospital of Hunan Normal University). Two groups (the control and Cri-Pt-CaFe_PB_ groups) of mice were treated with 0.9% NaCl and Cri-Pt-CaFe_PB_ nanozyme (100 μg/mL), respectively. The weights of the mice were recorded for 14 and 30 d after intravenous (i.v.) injection. The serum samples were collected and stored at -80 °C. All the organs were fixed in formalin, sectioned in sequence and stained with hematoxylin and eosin (H&E) for histological examination.

### Detection of hematological indicators

All hematological parameters, including aspartate aminotransferase (AST), alanine aminotransferase (ALT), blood urea nitrogen (BUN), creatinine (CRE), interleukin-1β (IL-1β), interleukin-6 (IL-6), and tumor necrosis factor-α (TNF-α), were assessed via commercially available reagent kits in this study. Testing was conducted in accordance with the manufacturer's recommended protocols.

### Establishment of a Mouse Model of SAP

All the mice (ICR, 6 weeks old, female) were randomly fasted for approximately 12 h prior to the experiments. Two groups of mice (SAP and Cri-Pt-CaFe_PB_) were treated with an intraperitoneal injection of Cerulein (CER, 50 μg/kg/h, 7 times). For the final injection, the mice received an intraperitoneal injection of lipopolysaccharide (LPS, 10 mg/kg, once). The mice of the control group were treated with 0.9% NaCl (n = 3).

### *In vivo* biodistribution and pharmacokinetics

To study the *in vivo* biodistribution of Cri-Pt-CaFe_PB_, a fluorescent molecule indocyanine green (ICG) was used to label Cri-Pt-CaFe_PB_ nanoparticles. The ICG-labeled Cri-Pt-CaFe_PB_ nanoparticles were formed by adding polyetherimide (PEI, 2 mg), ICG (5.0 mg/mL), and Cri-Pt-CaFe_PB_ (1.0 mg/mL) through simple stirring for 24 h. LPS-pretreated mice (SAP group) and healthy mice (control group) were injected with the same dosage of ICG-labeled Cri-Pt-CaFe_PB_ through tail vein, respectively. At different intervention time points (0, 1, 2, 4, 6, 8, 12, 24, 48 and 72 h), the mice in the two groups were analyzed by fluorescence imaging with an IVIS *in vivo* imaging system (Lumina XR series, PerkinElmer). Major organs (heart, liver, spleen, lung, kidney, and pancreas) and blood samples were collected at various time points for *ex vivo* imaging. Moreover, the pharmacokinetics was assessed by injecting 1 mg/kg of Cri-Pt-CaFe_PB_ into healthy* ICR* mice via tail vein. After administration at 0.5, 1, 2, 4, 6, 8, 12 ,24, and 48 h, the blood samples (100 µL) and pancreatic tissue were collected, and the Fe concentration in blood and pancreatic tissue was then determined by ICP-MS, and the half-life was calculated by noncompartmental analysis with Phoenix WinNolin software version 6.4 (Certera Inc., Princeton, N.J.) to evaluate the pharmacokinetics of Cri-Pt-CaFe_PB_.

### H&E and immunohistochemical (IHC) staining

The organ tissues were fixed with paraformaldehyde (4%), embedded in paraffin and sliced. The 4-μm deparaffinized slices were dyed with H&E to observe their histological features. Immunofluorescence staining was performed to detect M1 and M2 macrophages. Firstly, the pancreas sample sections (4 μm) were subjected to dewaxing, hydration, antigen extraction, goat serum blocking. Then, the sections were incubated with the corresponding antibodies, including anti-iNOS (1:200), anti-Arg-1 (1:200), and anti-F4/80 (1:1000). Finally, the imaging analysis was performed using a Leica DM8 B microscope.

### Protein immunoblotting

To detect the expression changes of CD86 and Arg-1, total protein from the pancreas were extracted with a cocktail-liked RIPA lysis buffer containing protease and phosphatase inhibitor.

### Statistical methods

In this study, the experimental data have been statistically analyzed via GRAPHPAD software, and statistical significance has been determined by the Mann-Whitney test (*p* < 0.05). Differences have been considered statistically significant if *p* value was not greater than 0.05. Standard notation has been derived as follows: *P ≤ 0.05, **P ≤ 0.01, ***P ≤ 0.001, and *ns* = not significant.

## Results and Discussion

### Synthesis and characterization of Cri-Pt-CaFe_PB_

**Figure 1:**
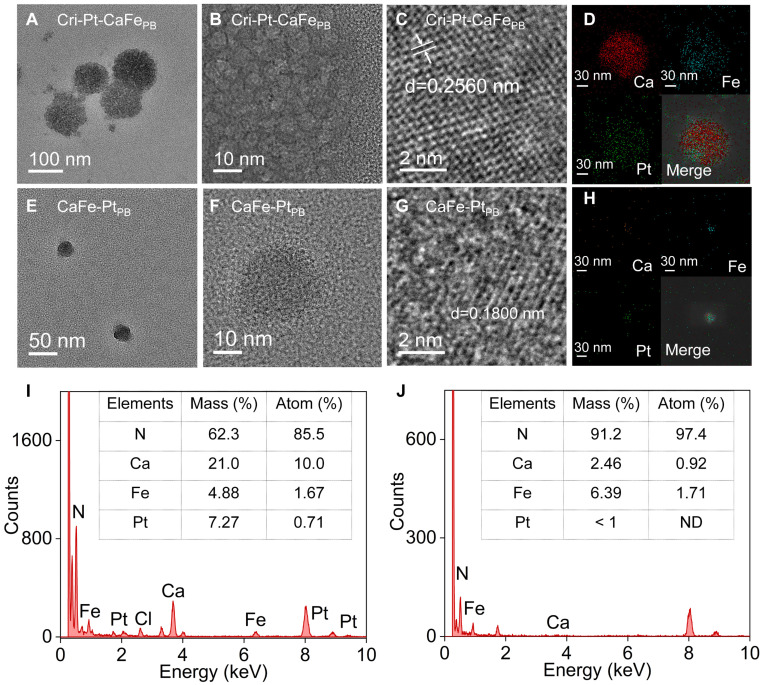
**Surface morphology of synthesized nanomaterials.** TEM **(A)** with its enlarged part **(B)**, HR-TEM **(C)**, and HAADF-STEM **(D)** with Ca, Fe, Pt, and merge images of Cri-Pt-CaFe_PB_. TEM **(E)** with its enlarged part **(F)**, HR-TEM **(G)**, and HAADF-STEM **(H)** with Ca, Fe, Pt, and merge images of CaFe-Pt_PB_. Corresponding EDS spectra of Cri-Pt-CaFe_PB_ **(I)** and CaFe-Pt_PB_ **(J)**, and the Table in the Figure 1I and J is thin film standardless quantitative analysis, respectively.

Calcium chloride (CaCl_2_) and potassium ferricyanide (K_3_[Fe(CN)_6_]) were used as precursors to prepare a calcium hexacyanoferrate Prussian blue-like (CaFe_PB_) nanomaterial via a freeze-thaw method in a medium of PVP, which was served as both reducing- and dispersing-agent 30, 32. TEM image in Figure S1 reveals that the PEG-modified CaFe_PB_ exhibits a spheroidal ultrastructure with an average size of 5.0 ± 1.2 nm (n = 65). A platinum(IV) substance can be converted into a FAD-approved cisplatin(II) drug 28, 29, 33, making it an ideal connector for grafting onto the CaFe_PB_ surface because of its rich chemical composition and excellent biosafety 29. By altering the sequence of Pt doping and PEG modification, the controlled synthesis of two Prussian blue analogs with distinct structures were achieved. As shown in Figure 1a, the surface of CaFe_PB_ is modified with PEG and then dopped with chloroplatinic acid (H_2_PtCl_6_∙6H_2_O) to obtain cisplatin- and Pt(IV)-conjugated hexacyanoferrate Prussian blue analogs (Cri-Pt-CaFe_PB_). The product retaines a regular spherical morphology, but its average particle size is increased to approximately 105 nm (Figure 1A). The increased size of Cri-Pt-CaFe_PB_ can be attributed to coordination coupling between Pt and the carboxyl group on the surface of the CaFe_PB_ nanomaterial. Moreover, the individual TEM image in Figure 1B reveals the localized microstructure of Cri-Pt-CaFe_PB_, showing that it is a spherical nanomaterial formed by the orderly arrangement and assembly of ultrastructural nanospheres identical to CaFe_PB_. This structure confirms that Pt(IV) can act as a connector to link ultrastructural CaFe_PB_ (~ 5 nm), forming Cri-Pt-CaFe_PB_ (~105 nm). Notably, many cracks are observed among the ultrastructural nanospheres (Figure 1A-B). These surface features promote the exposure of catalytically active sites, thereby enhancing effective interactions with target molecules and thus significantly improving the catalytic efficiency of the Cri-Pt-CaFe_PB_. This result is consistent with the findings reported by Li *et al* 34. Additionally, the high-resolution TEM image in Figure 1C reveals distinct lattice fringes in the Cri-Pt-CaFe_PB_ nanomaterials, with an interplanar spacing of 0.26 nm. Elemental mappings (Figure 1D) present homogeneous distribution of Ca (red), Fe (blue), and Pt (green) within the Cri-Pt-CaFe_PB_ nanomaterial, further proving that Pt is integrated into the nanosphere through coordination coupling rather than being deposited on the surface.

In contrast, a modified synthesis approach was employed using a one-step freeze-thaw method to produce platinum-doped calcium hexacyanoferrate Prussian blue analogs (CaFe-Pt_PB_) with spherical nanostructures. The average particle size of these nanomaterials was approximately 20 nm (Figure 1E). The TEM image of the individual nanoparticle shown in Figure 1F reveals that the surface of the CaFe-Pt_PB_ nanosphere is smooth, with a lattice spacing of 0.18 nm (Figure 1G). The HAADF-STEM mapping combined with EDS analysis reveals that the content of Pt in Cri-Pt-CaFe_PB_ (Figure I) is higher than that in CaFe-Pt_PB_ (Figure J). Furthermore, the content of Ca elemental in Cri-Pt-CaFe_PB_ (stepwise method, Section “Preparation of Cri-Pt-CaFe_PB_”) is also higher than that in CaFe-Pt_PB_ (one-step method, Section “Preparation of CaFe-Pt_PB_”), which is mainly due to the differences in the introduction of Ca^2+^, metal ion coordination competition and kinetic control between the two methods. The stepwise method is conducive to the directional introduction of Ca^2+^, while the one-step method is limited by multi-component competition, which may lead to insufficient Ca^2+^. These findings suggest that the structure of Pt-doped Prussian blue nanomaterials can be precisely controlled by optimizing the synthesis process.

To study the effects of various components on the structure of the nanomaterials, PtFe_PB_ and PtCa_PB_ bimetallic nanocomposites were prepared via the freeze-thaw method (Figure S2-S3). As shown in Figure S2, the PtCa_PB_ nanomaterials exhibit an irregular morphology, whereas the PtFe_PB_ nanomaterials feature monodisperse nanocages with a particle size of approximately 20 nm (Figure S3). It can be seen that the chemical composition and synthesis process play crucial roles in the structure and properties of the nanomaterials.

**Figure 2:**
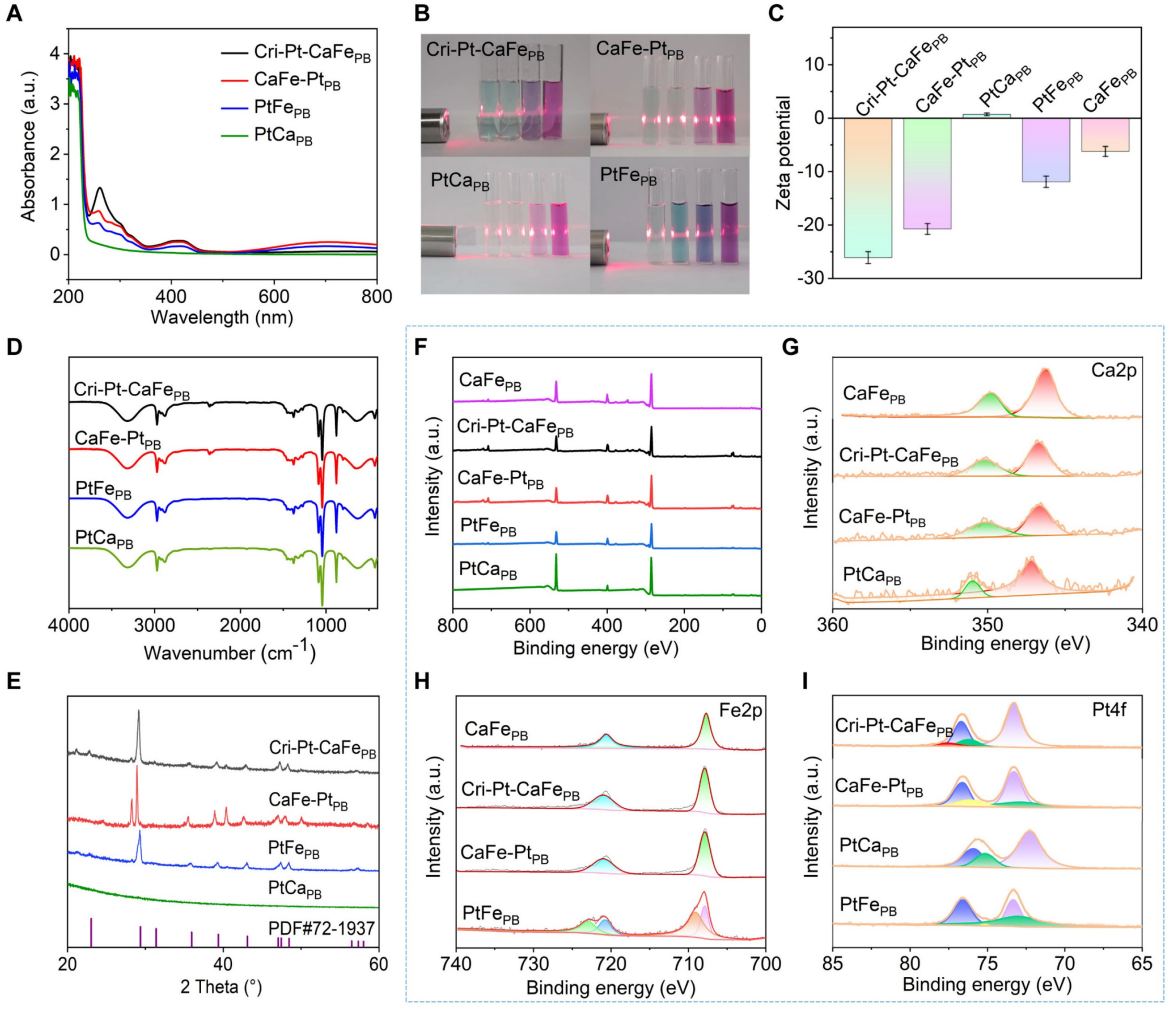
**Structural characterization of synthesized nanomaterials.** UV-visible absorption spectra in water **(A)** of various nanomaterials, and their Tyndall effects in different medium solutions **(B)** of H_2_O, PBS, RPMI-1640, and DMEM (Inset of every four different color cells, from left to right), respectively. Corresponding Zeta potentials **(C)**, FT-IR spectra **(D)**, and XRD patterns **(E)**. XPS full spectra **(F)** and corresponding Ca2p **(G)**, Fe2p **(H)**, and Pt4f **(I)** spectra, respectively.

The as-synthesized Cri-Pt-CaFe_PB_, CaFe-Pt_PB_, PtCa_PB_, and PtFe_PB_ nanomaterials were further characterized using UV-vis-NIR spectroscopy. As illustrated in Figure 2A, the absorption peaks of all four nanomaterials are appeared at 260 nm and 400-450 nm, among which the absorption peak of Cri-Pt-CaFe_PB_ is the highest, which may be attributed to the fact that Cri-Pt-CaFe_PB_ contains the most Pt ions. The four nanomaterials exhibit obvious Tyndall effects in various physiological/medium solutions, including aqueous solutions, PBS, PMMI-1640, and DMEM (Figure 2B). Besides the Tyndall effect experiment, the dispersion and long-term stability of Cri-Pt-CaFe_PB_ in an aqueous solution were also monitored by dynamic light scattering (DLS) for a period of 4 weeks (Figure S4). Figure S4A shows a uniform size distribution centered on a peak of about 105 nm, and almost unchanged in 4 weeks (Figure S4B), indicating its long-term stability in aqueous solution. Zeta potential results in Figure 2C reveal that the Cri-Pt-CaFe_PB_ nanomaterial is negatively charged and exhibits the best stability among the tested samples. The zeta potential value of Cri-Pt-CaFe_PB_ (-26.16 mV) is the lowest compared with those of CaFe-Pt_PB_ (-20.79 mV), PtCa_PB_ (0.68 mV), PtFe_PB_ (-11.96 mV), and CaFe_PB_ (-6.26 mV). As illustrated in Figure 2D, no significant difference can be observed in the FT-IR spectra of Cri-Pt-CaFe_PB_, CaFe-Pt_PB_, PtCa_PB_, and PtFe_PB_, which is consistent with the results reported in the literature 30, indicating that the incorporation of trace amounts of Pt does not alter the characteristic functional groups of CaFe_PB_. X-ray diffraction (XRD) data in Figure 2E reveals that Cri-Pt-CaFe_PB_ exhibits a calcite-type structure, with its ion arrangement following the compact packing principle of PDF#72-1937, whereas the CaFe-Pt_PB_ nanomaterial may have a heterogeneous phase, indicating that Pt interferes with the incorporation of Ca^2+^. This result is consistent with EDS data (Figure 1I-J). Furthermore, the chemical composition and elemental valence state of four nanomaterials were investigated using X-ray photoelectron spectroscopy (XPS), with their full spectra shown in Figure 2F. For all samples, the Ca2p peaks exhibit two characteristic peaks of Ca2p3/2 and Ca2p1/2 at 346.0 and 350.0 eV, respectively, with an intensity ratio of approximately 2:1, revealing the existence of Ca(II) in the as-synthesized Cri-Pt-CaFe_PB_, CaFe-Pt_PB_, and PtCa_PB_ (Figure 2G). The two peaks of Fe2p3/2 (707.9 eV) and Fe2p1/2 (721.1 eV) in Figure 2H confirm the presence of Fe^3+^ in the successfully prepared CaFe_PB_, Cri-Pt-CaFe_PB_, and CaFe-Pt_PB_. Notably, the Fe2p spectrum of PtFe_PB_ shows complex multiple splitting, which can be attributed to the high-spin compounds of PtFe_PB_. The spectra of Pt4f matched those of Pt(II) and Pt(IV) in all of the four nanomaterials, with peaks at 73.1 eV and 76.2 eV, respectively (Figure 2I). The comparable peak areas further validate the successful preparation of Cri-Pt-CaFe_PB_ nanomaterials conjugated with cisplatin and Pt(IV).

### Multienzyme-like activities

**Figure 3:**
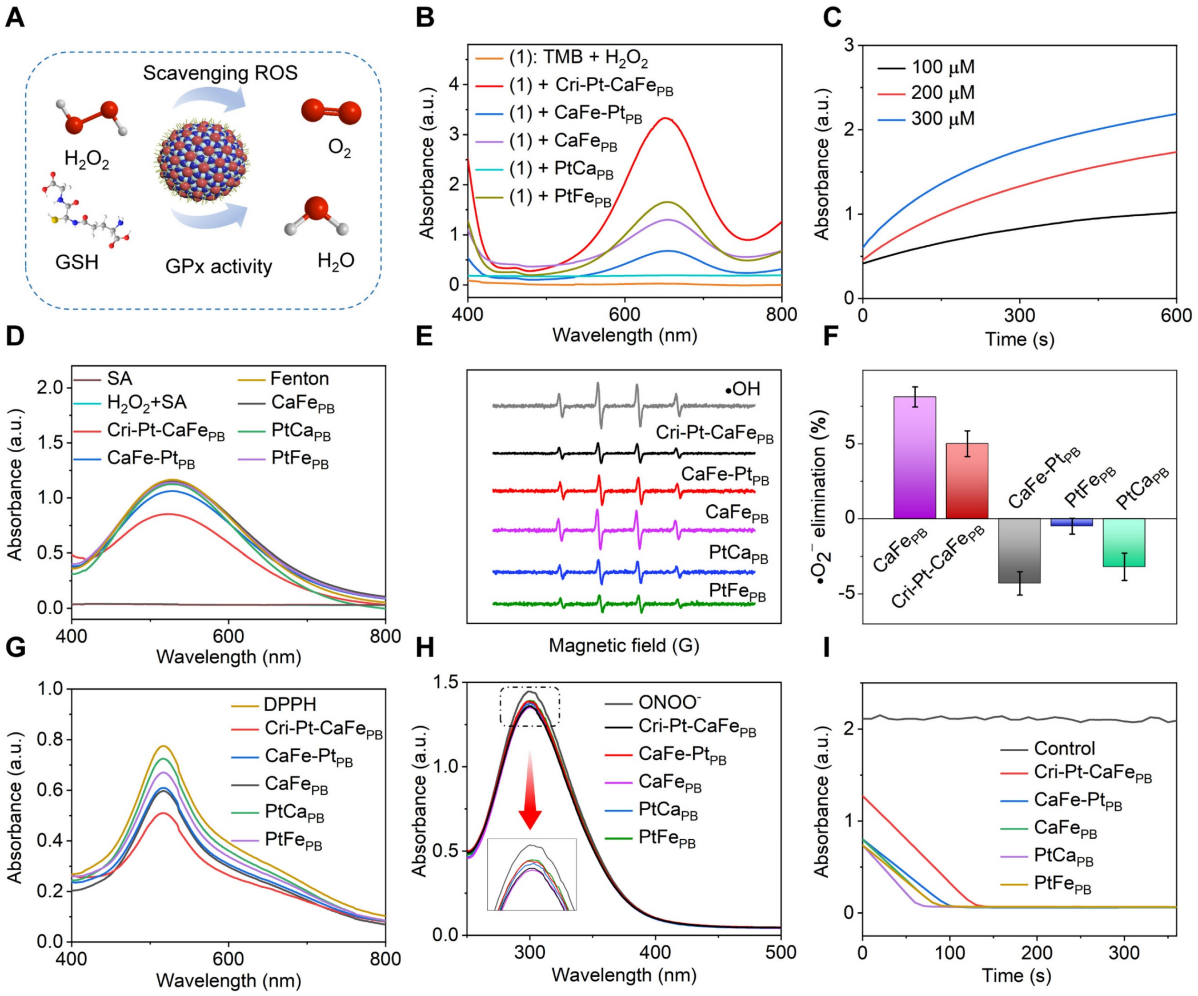
**Multienzyme-like activities and antioxidant properties of synthesized nanomaterials. (A)** Schematic illustration of the ROS scavenging effect of Cri-Pt-CaFe_PB_ on the basis of its multienzyme-like activity. **(B)** UV-vis spectra of synthesized nanomaterials with POD-like activity. **(C)** Absorption values of Cri-Pt-CaFe_PB_ solutions upon addition of TMB and different concentrations of H_2_O_2_ changed with the time. **(D)** UV-vis spectra for •OH elimination activity with various nanozymes and the Fenton reagent. **(E)** EPR spectra of •OH elimination. **(F)** SOD-like activities of the nanomaterials. **(G)** DPPH- and **(H)** ONOO- scavenging abilities of the nanozymes. **(I)** GPx-like activities of synthesized nanomaterials. The concentration of each nanomaterial was 200 µg/mL.

The pathogenesis of SAP is complex and multifaceted, with oxidative stress induced by ROS in most cases. Therefore, targeted elimination of endogenous ROS is a potential therapeutic strategy for inflammatory diseases 35. In this study, three representative ROS (H_2_O_2_, •OH, and •O^2-^) and RNS (DPPH• and ONOO^-^) were chosen to explore the different natural enzyme-mimicking activities and RO/NSs-scavenging capabilities of the nanomaterials *in vitro* (Figure 3A). Peroxidase (POD) is a crucial endogenous reactive oxygen scavenger that degrades H_2_O_2_ into H_2_O. Using TMB with H_2_O_2_ as the catalytic substrate, the POD enzyme activities of these nanomaterials were investigated by means of changes in absorption spectra. Compared with CaFe-Pt_PB_, PtCa_PB_, PtFe_PB_, and CaFe_PB_, Cri-Pt-CaFe_PB_ presents the highest absorbance at 652 nm (Figure 3B), proving its greater peroxidase activity, being able to effectively remove hydrogen peroxide (H_2_O_2_). The POD-like catalytic capacity of Cri-Pt-CaFe_PB_ is enhanced with increasing H_2_O_2_ concentrations (Figure 3C). In the meantime, the oxidase-like activity of these nanomaterials was also examined (Figure S5). The characteristic peak at a wavelength of 652 nm can be negligible, revealing that those five nanomaterials have almost no oxidase-like activity. To assess the ability of nanomaterials to remove •OH, Fenton reagent was used to generate •OH, and salicylic acid was oxidized by •OH via monitoring its characteristic absorption peak at 510 nm. Upon addition of Cri-Pt-CaFe_PB_, the absorption peak is significantly decreased, showing its strongest •OH scavenging activity (Figure 3D). Consistently, the electron paramagnetic resonance (EPR) results in Figure 3E show a reduction in the characteristic 1:2:2:1 peak of •OH produced by the Fe^2+^/H_2_O_2_ Fenton system with the addition of the nanozymes.

The superoxide dismutase (SOD)-like activity of various nanomaterials was evaluated using the pyrogallol autoxidation method 36. Under weakly alkaline conditions, pyrogallol undergoes autoxidation to yield a yellow solution with a characteristic absorption peak at 325 nm, allowing assessment of the SOD-like activities. As shown in Figure 3F, the superoxide anion (·O_2_^-^) clearance ratios are 8.12% for CaFe_PB_ and 5.0% for Cri-Pt-CaFe_PB_, respectively. In contrast, CaFe-Pt_PB_, PtCa_PB_ and PtFe_PB_ display negative clearance rates, potentially due to their promotion of pyrogallol autoxidation. To confirm the nanomaterials' general RNS scavenging capabilities, DPPH•, a stable nitrogen radical with abundant unpaired electrons, was employed. As shown in Figure 3G, the absorbance peak of DPPH• at 517 nm is decreased after treatment with different nanozymes, among which Cri-Pt-CaFe_PB_ is demonstrated as the most effective DPPH• scavenger. Furthermore, as shown in Figure 3H, the ONOO- scavenging abilities of the five nanomaterials show almost no significant differences. The activity of GPx, which can reduce toxic peroxides to non-toxic compounds by converting reduced glutathione (GSH) into oxidized glutathione (GSSG), was also investigated. This reaction is reversed by nicotinamide adenine dinucleotide phosphate (NADPH) 35. As shown in Figure 3I, the absorbance of NADPH, reduced by GSH, is significantly decreased within 110 s. These findings indicate that Cri-Pt-CaFe_PB_ exhibits superior multienzyme activity, including POD, SOD, and GPx-like ones, and possesses a broader antioxidant capacity compare to CaFe-Pt_PB_, PtCa_PB_, PtFe_PB_ and CaFe_PB_. This makes Cri-Pt-CaFe_PB_ a promising candidate for eliminating various oxidative damage and providing anti-inflammatory effects both *in vivo* and *in vitro*.

### DFT calculations of Cri-Pt-CaFe_PB_

**Figure 4:**
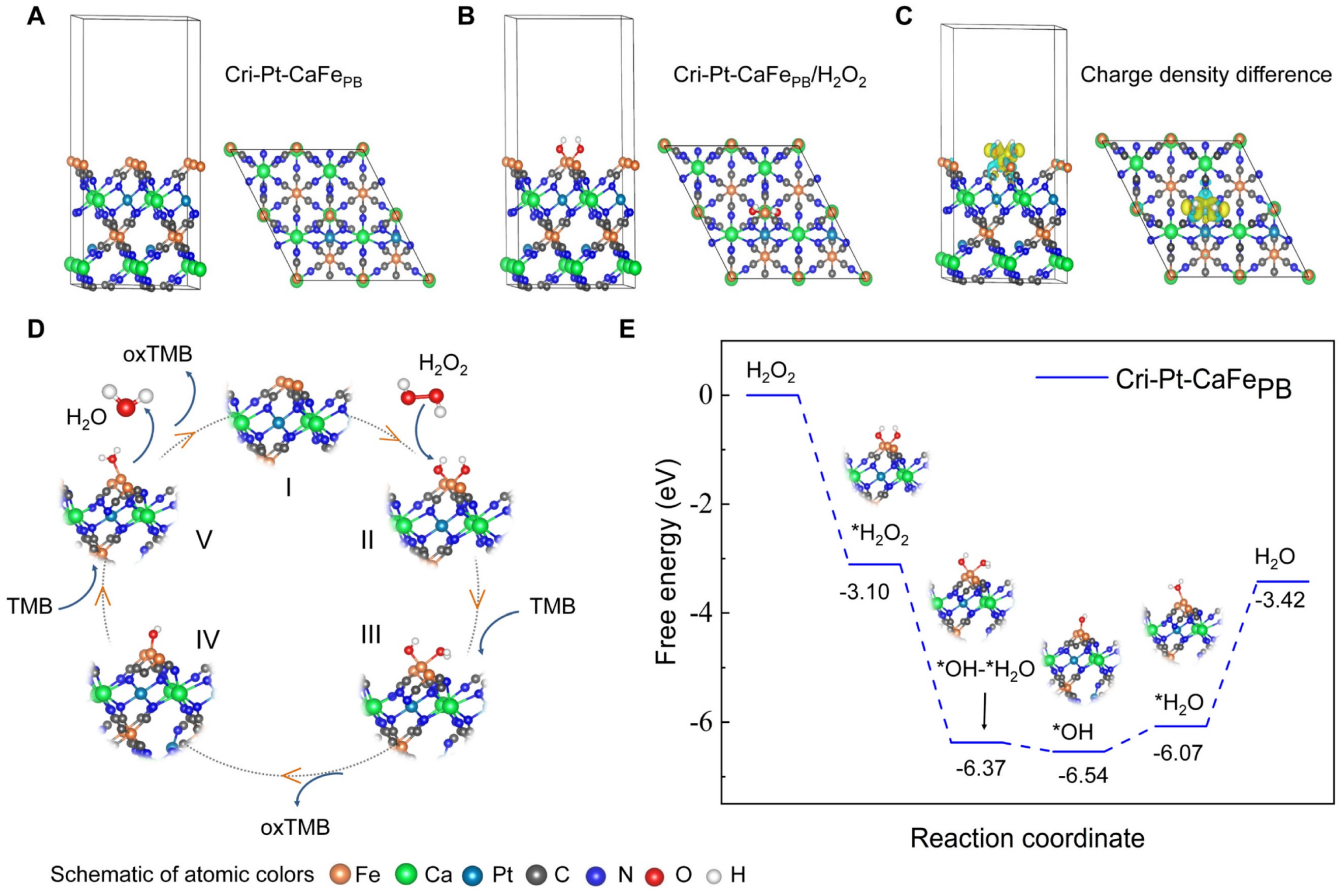
**DFT simulation on the POD enzyme-like activity of Cri-Pt-CaFe_PB_. (A)** Cri-Pt-CaFe_PB_ (111) surface structure. **(B)** Stable configurations of H_2_O_2_ adsorbed on the Cri-Pt-CaFe_PB_ (111) surface. **(C)** Charge density difference of Cri-Pt-CaFe_PB_. **(D)** Schematic representation of the reaction route to obtain a catalytic cycle for TMB oxidation and **(E)** its free energy changes.

Given the exceptional ROS scavenging abilities and diverse enzyme-mimicking activities of Cri-Pt-CaFe_PB,_ including POD, SOD, and GPx, DFT calculations were conducted to investigate the intrinsic catalytic mechanism of H_2_O_2_ decomposition on the surface of Cri-Pt-CaFe_PB_. The detailed calculation process is given in the Supporting Information. A structural model of Cri-Pt-CaFe_PB_ was firstly constructed and optimized using DFT based on the structural composition revealed by TEM (Figure 1E-I) and the crystal structure confirmed by XRD patterns (Figure 2E). Figure 4A presents a single crystal structure from the (111) crystal plane of Cri-Pt-CaFe_PB_ after structural optimization, where the left panel is a side view of the optimized structure and the right panel is a top view. It can be seen from Figure 4A that the structure of Cri-Pt-CaFe_PB_ is formed by the connection of Fe atoms and Ca atoms through the -C-N- bonds, and Pt atoms are embedded in the lattice gaps through coordination with N atoms. This structural model lays the foundation for understanding the catalytic properties and ROS-scavenging mechanisms of Cri-Pt-CaFe_PB_ at the atomic level. Figure 4B shows the optimized model of Cri-Pt-CaFe_PB_ bound with hydrogen peroxide (H_2_O_2_), where the Fe atom in the Cri-Pt-CaFe_PB_ crystal binds to two oxygen atoms of H_2_O_2_. Moreover, the binding interaction between the model and H_2_O_2_ can be revealed by analyzing the difference in charge density (Figure 4C), where the yellow and blue regions indicate electron accumulation and electron depletion, respectively. From Figure 4C, it can be seen that there is a large yellow region between the oxygen atom in H_2_O_2_ and the Fe atom in the Cri-Pt-CaFe_PB_ model, confirming the formation of a strong covalent bond between the two atoms. The catalytic pathway and corresponding Gibbs free energy for simulating the elimination of H_2_O_2_ by POD on the surface of Cri-Pt-CaFe_PB_ are depicted in Figure 4D-E. And the optimized structure of intermediate in catalytic pathway was shown in Figure S6. Firstly, H_2_O_2_ adsorbs onto the Fe atom on the Cri-Pt-CaFe_PB_ surface, followed by the decomposition of the O-O bond into two •OH groups, and then binds to the Fe site 37. This step is a downhill process due to the strong adsorption capacity of H_2_O_2_ on the Cri-Pt-CaFe_PB_ surface. The hydrogenation process involves transferring hydrogen atoms from the amino group of TMB to the hydroxyl group on the Fe atom via protonation coupling 38. The initial hydrogenation of converting *OH-*OH to *OH-*OH-H is exothermic with a free energy of 3.27 eV. This is followed by an endothermic step of 0.17 eV, where *OH-*OH-H is converted to *OH-*H_2_O, resulting in the formation of oxidation state TMB (oxTMB). The second hydroxyl group adsorbed at the Fe site regenerates the active sites on the Cri-Pt-CaFe_PB_ surface, thus completing the catalytic cycle. In summary, Cri-Pt-CaFe_PB_ catalyzes hydrogen oxidation with a low energy barrier, showcasing its high POD-like activity. These DFT data align well with the experimental findings on the POD-like activity of Cri-Pt-CaFe_PB_ (Figure 3).

### Biosafety assessment of Cri-Pt-CaFe_PB_

The biosafety assessment of Cri-Pt-CaFe_PB_* in vitro* and *in vivo* is crucial for their potential clinical applications. Firstly, pancreatic exocrine cells (AR42J) were used to evaluate the cytotoxicity of Cri-Pt-CaFe_PB_ *in vitro* via a CCK-8 assay. As can be seen from Figure S7 and Table S1, at different time points (1, 6, 24, and 48 h), the cell survival rate remains above 93% after co-incubation with Cri-Pt-CaFe_PB_ at a dose concentration below 400 µg/mL, indicating the excellent biosafety of the Cri-Pt-CaFe_PB_ *in vitro*. Secondly, the protective effect of Cri-Pt-CaFe_PB_ on LPS-induce RAW264.7 (macrophages) cells was observed. Compared with SAP group, the cell survival rate after Cri-Pt-CaFe_PB_ treatment was significantly improved, which was close to that of the control group when the concentration of Cri-Pt-CaFe_PB_ exceeded 100 µg/mL (Figure S8). Therefore, the concentration of of Cri-Pt-CaFe_PB_ was selected as 200 µg/mL for subsequent cellular and mice experiments.

**Figure 5:**
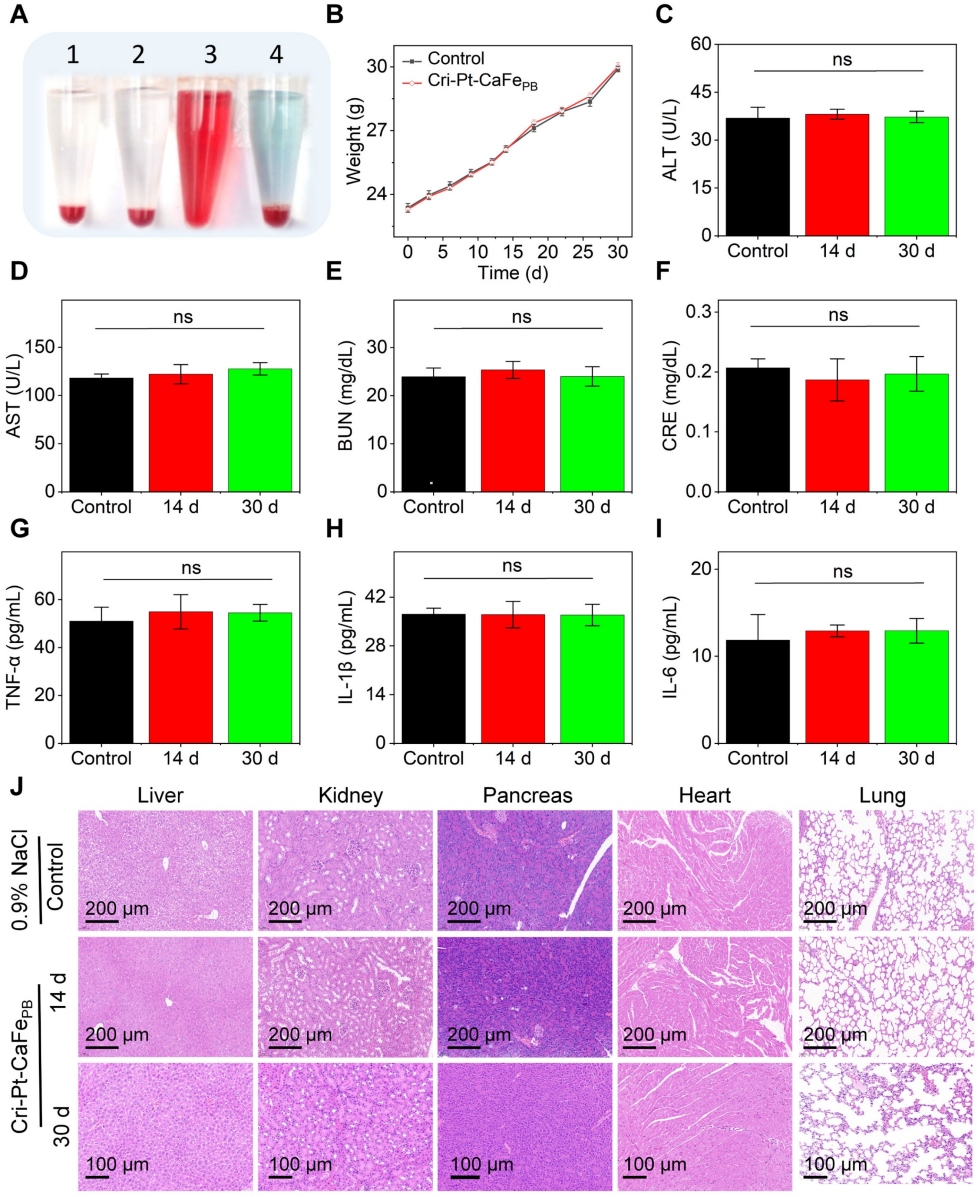
** Biosafety assessment of Cri-Pt-CaFe_PB_. (A)** Photographs of RBCs in hemolysis reactions incubated for 4 h with different solutions: PBS (1), 0.9% NaCl (2), H_2_O (3), and Cri-Pt-CaFe_PB_ (4). **(B)** Mice weight changes with 0.9% NaCl (control) and Cri-Pt-CaFe_PB_ treated for 30 d (n = 3). Mice serum analysis of ALT **(C)**, AST **(D)**, BUN **(E)**, CRE **(F)**, TNF-α **(G)**, IL-1β **(H)**, and IL-6 **(I)** subjected to different treatments. **(J)** H&E staining of mice organs, such as liver, kidneys, pancreas, heart, and lungs, treated with Cri-Pt-CaFe_PB_ over 14 and 30 d. *ns* means not significant.

To further study the biocompatibility and of Cri-Pt-CaFe_PB_ *in vivo*, healthy mice subjected to different treatments were injected through tail vein. As shown in Figure 5A, hemolysis reaction occurs only in red blood cells (RBCs) treated with H_2_O, while the erythrocytes remain intact when treated with Cri-Pt-CaFe_PB_, PBS, or 0.9% NaCl solution, demonstrating good blood compatibility of the Cri-Pt-CaFe_PB_. In addition, there is no significant difference in body weight between the control group (0.9% NaCl) and the Cri-Pt-CaFe_PB_ treated group over 30 d as shown in Figure 5B, suggesting that Cri-Pt-CaFe_PB_ exhibits excellent metabolic compatibility and lacks *in vivo* biotoxicity. Liver and kidney function indicators are critical for evaluating the physiological toxicity of nanodrugs 39. The long-term toxicity and reproductive toxicity *in vivo* of Cri-Pt-CaFe_PB_ were also investigated by measuring the contents of liver and kidney function indicators in different serum collected from healthy mice at 1 to 30 d post-injection. Several hematological parameters, including ALT (Figure 5C), AST (Figure 5D), BUN (Figure 5E), and CRE (Figure 5F), as well as inflammatory cytokines such as TNF-α (Figure 5G), IL-1β (Figure 5H), and IL-6 (Figure 5I), were evaluated. It is confirmed that the levels of liver and kidney function indicators and inflammatory factors in the Cri-Pt-CaFe_PB_-treated group are essentially identical to those in the healthy control group after 14 and 30 d of intervention treatment, confirming that the Cri-Pt-CaFe_PB_ possess long-term biocompatibility and do not induce inflammation *in vivo*. Moreover, H&E staining images reveal no significant histological or morphological changes in the pancreas, liver, kidneys, heart, or lungs in either the Cri-Pt-CaFe_PB_ treated group compared with the control group at 14 and 30 d (Figure 5J). And the biocompatibility evaluation results of Cri-Pt-CaFe_PB_ were compared with those of other PB-based nanozymes in Table S2, indicating that the Cri-Pt-CaFe_PB_ nanozyme exhibits long-term biosafety and negligible systemic toxicity *in vitro* and *in vivo* within the dosage range.

### Anti-inflammatory effect of Cri-Pt-CaFe_PB_ *in vitro*

**Figure 6:**
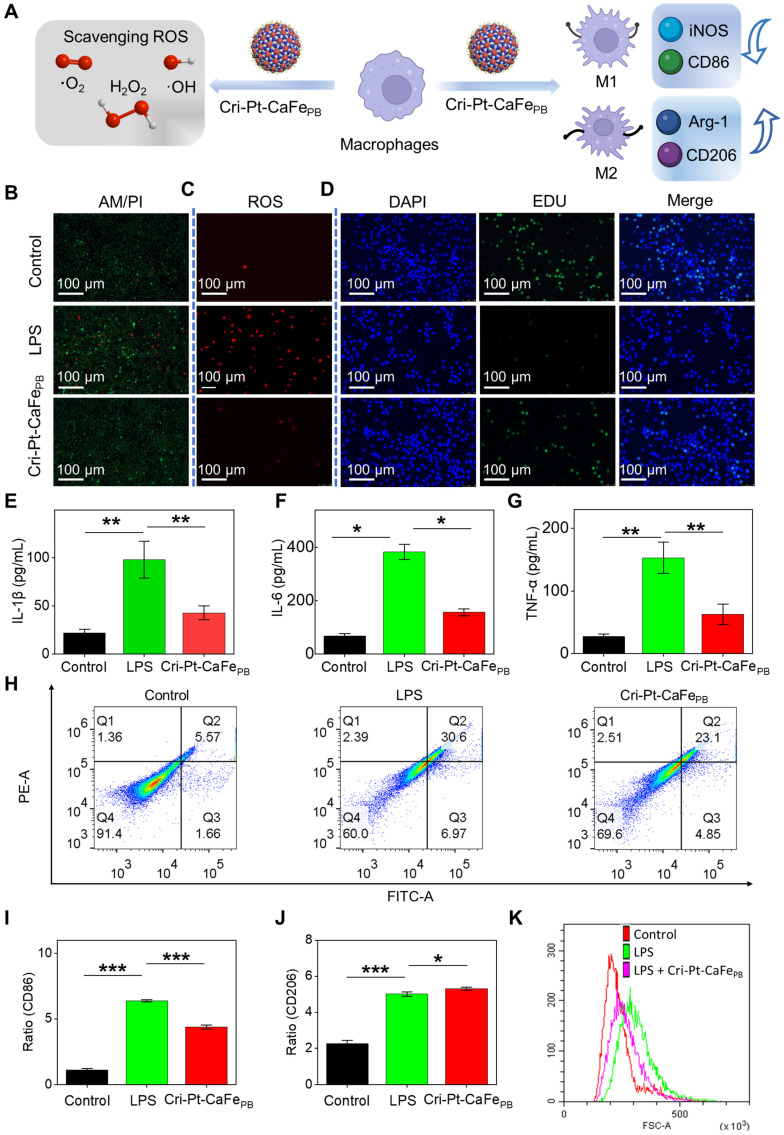
** Anti-oxidative and anti-inflammatory effect of Cri-Pt-CaFe_PB_ *in vitro*. (A)** Schematic illustration of therapeutic mechanism of Cri-Pt-CaFe_PB_ nanozyme *in vitro* at cellular level. CLSM images for cells in three groups (Control, LPS, and LPS+Cri-Pt-CaFe_PB_) stained with Calcein AM/PI **(B)**, DCFH-DA **(C)**, and EdU **(D)**, and the scale bar is 100 μm. The expression of inflammatory factors of IL-1β **(E)**, IL-6 **(F)**, and TNF-α **(G)** in cells subjected to different treatments. **(H)** Quantification of CD86 (an M1 marker) and CD206 (an M2 marker) expression in cells by flow cytometry. Change the ratios of CD86 **(I)** and CD206 **(J)** in the three groups. **(K)** Intracellular ROS levels in RAW 264.7 cells stained with DCF-DA. ***P < 0.001, **P < 0.01, *P < 0.05.

Macrophage polarization plays a decisive role in the pathogenesis of SAP 40. Particularly, macrophages exhibit different phenotypes with distinct functions in SAP, for example, M1 macrophages release proinflammatory cytokines and chemokines, exacerbating the development of inflammatory diseases, while M2 macrophages secrete anti-inflammatory cytokines, promoting the resolution and recovery of inflammatory 40-43. ROS scavenging is a key factor in regulating macrophage differentiation 44, 45. Therefore, facilitating the transformation of M1- into M2-subtype through ROS clearance is highly beneficial for the treatment of inflammatory diseases. To further elucidate the therapeutic mechanism of Cri-Pt-CaFe_PB_ at cellular level, the endogenous ROS levels and macrophage polarization in LPS-induced RAW264.7 cells were evaluated by the treatment with Cri-Pt-CaFe_PB_ (Figure 6A). The cells were divided into three groups, namely, the control group, the LPS-treated group, and the Cri-Pt-CaFe_PB_-treated group. As shown in Figure 6B, a large number of dead cells (indicated by red fluorescence) are observed after LPS treatment via AM-PI staining. However, cell death caused by LPS is significantly reduced when the cells are cocultured with Cri-Pt-CaFe_PB_, indicating that nanomedicine can effectively protect the cells from oxidative damage. Meanwhile, the immunofluorescence images shown in Figure 6C reveal a marked reduction in intracellular ROS levels after Cri-Pt-CaFe_PB_ treatment in LPS-treated cells, along with restored cell proliferation activity, highlighting the ROS-clearing capability of the nanozymes *in vitro*. And the antioxidant capacity of Cri-Pt-CaFe_PB_ is also confirmed by the immunofluorescence imaging with EDU staining (Figure 6D). The expression levels of inflammatory mediators associated with SAP pathophysiology, including TNF-α, IL-1β and IL-6, were analyzed by extracting the cell supernatant. As show in Figure 6E-G, IL-1β, IL-6, and TNF-α were highly expressed in SAP group, indicating that SAP model has been successfully established after LPS treatment for 24 h, which is consistent with the pathological characteristics of inflammation models reported in other literatures 31. After incubation with Cri-Pt-CaFe_PB_, the levels of these inflammatory factors were significantly reduced, indicating effective inhibition of the inflammatory cytokine storm by Cri-Pt-CaFe_PB_.

In the early stage of SAP, macrophage polarization is enriched in the pancreas, with the balance between its subtypes of pro-inflammatory M1 and anti-inflammatory M2), determining the severity of inflammation 46-48. To distinguish the polarization of M1 and M2 subtypes of macrophages *in vitro*, we evaluated the trend of the macrophage polarization in pancreatitis before and after nanozyme treatment using CD86 (an M1 marker) and CD206 (an M2 marker) proteins. As shown in Figure 6H, flow cytometry results reveal increased CD86 expression in the LPS-induced group, indicating polarization toward the M1 phenotype during the pathogenesis of SAP. However, in the Cri-Pt-CaFe_PB_ treatment group, CD206 expression is upregulated and CD86 expression is reduced, suggesting a shift toward M2 macrophage polarization with anti-inflammatory effects. Moreover, the percentages of CD86 protein expression are 1.01%, 6.38%, and 4.24% for the control, LPS, and nanozyme groups, respectively, while the percentages of CD206 expression are 2.46%, 5.13% and 5.22%, respectively (Figure 6I). The DCF-DA staining analysis of ROS levels in RAW264.7 cells after different treatments is shown in Figure 6J. Compared with the LPS group, the ROS levels in the nanozyme-treated group is decreased, as indicated by the left toward shift in the peak, which is consistent with the results shown in Figure 6C. The above experimental results demonstrate that Cri-Pt-CaFe_PB_ with ROS-clearing activity can regulate the polarization of macrophages by modulating the M1/M2 ratio, thereby achieving precise treatment of SAP at the cellular level.

### *In vivo* biodistribution and pharmacokinetic of Cri-Pt-CaFe_PB_

To further validate the therapeutic potential of Cri-Pt-CaFe_PB_ *in vivo*, the biodistribution and pharmacokinetics of nanoparticles in mice was traced by the injection of ICG-labeled Cri-Pt-CaFe_PB_, followed by NIR fluorescence imaging analysis. Firstly, ICG fluorescent molecules were loaded into Cri-Pt-CaFe_PB_ nanoparticles by simple stirring, and the change of zeta potential in Figure S9 confirmed the successful anchoring of ICG on the Cri-Pt-CaFe_PB_ surface. Secondly, ICG-labeled Cri-Pt-CaFe_PB_ nanozyme was injected into SAP mice via tail vein, and the health mice was used as control.

**Figure 7:**
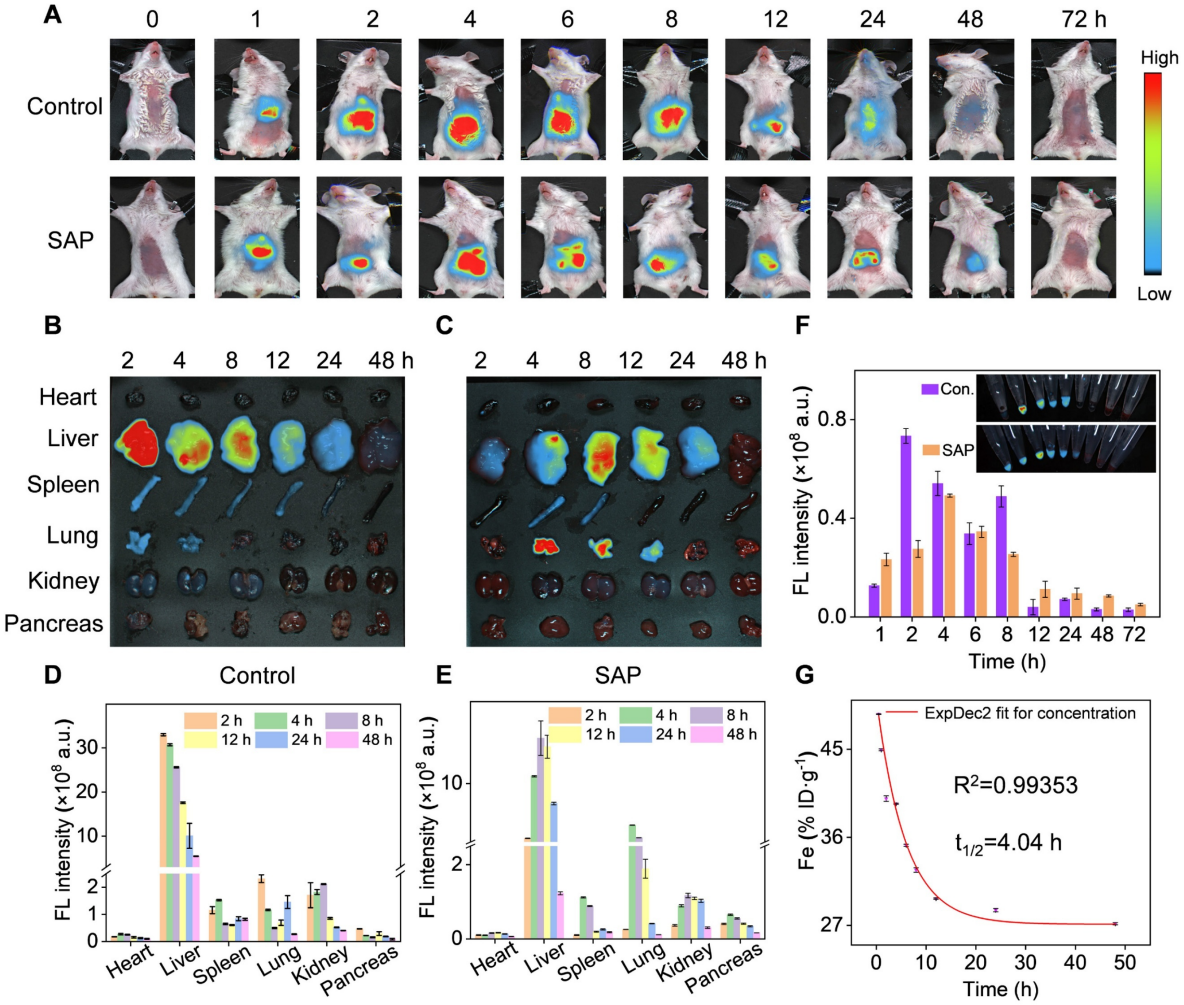
***In vivo* biodistribution and pharmacokinetic of Cri-Pt-CaFe_PB_**. **(A)** Fluorescence images of mice in control (healthy mice) and SAP group after injection with ICG-labeled Cri-Pt-CaFe_PB_ at various time points. *Ex vivo* fluorescence images of main organs in **(B)** control and **(C)** SAP groups at various time points. Corresponding fluorescence intensities of dissected major organs at various time points in **(D)** control and **(E)** SAP groups. **(F)** The blood fluorescence intensities of different groups over time, and the inset is the corresponding fluorescence image (Con. means control). **(G)** The pharmacokinetics of Fe element during blood circulation over time in healthy mice after injection of Cri-Pt-CaFe_PB_ (n = 3).

As depicted in Figure 7A, the intraperitoneal image of mice shows that ICG fluorescence appears at 1 h after injection, and gradually increases to the brightest level at 4 h for SAP group and 8 h for control group, then gradually weakened until disappeared, indicating that Cri-Pt-CaFe_PB_ can rapidly accumulate in SAP mice. Subsequently, the biodistribution of nanozyme in the main organs of mice at different time points after injection of ICG-labeled Cri-Pt-CaFe_PB_ was further explored. The* ex vivo* fluorescence images (Figure 7B-C) and corresponding fluorescence intensities (Figure 7D-E) reveal that ICG-labeled Cri-Pt-CaFe_PB_ mainly accumulates in the liver within 2 h, with less accumulation in the heart, spleen, lungs, and kidneys, indicating that the liver has a strong interception effect. The fluorescence imaging and corresponding fluorescence intensity of the blood of healthy mice collected at different time points have also explained the distribution and the blood circulation of ICG-labeled Cri-Pt-CaFe_PB_ in blood (Figure 7F). And the distribution of ICG-labeled Cri-Pt-CaFe_PB_ in mice shows a dose-dependent relationship (Figure S10).

Beyond biodistribution, we further explored the pharmacokinetic of Cri-Pt-CaFe_PB_ in blood and pancreas tissue by ICP-MS with Fe element as an index. As shown in Figure 7G, the Fe content in blood collected from healthy mice injected with Cri-Pt-CaFe_PB_ is stabilized at about 30 h, and the circulation half-lives (t_1/2_) is inferred to be 4.04 h. In addition, ICP-MS quantitative analysis the level of Fe in pancreatic tissue of the SAP group is significantly higher than that of the control group (Figure S11), which ensures the possibility of Cri-Pt-CaFe_PB_ providing treatment in inflammation. Therefore, all of the above results exhibit the superior delivery efficiency of Cri-Pt-CaFe_PB_, showing potential applications* in vivo*.

### *In vivo* therapeutic effect of Cri-Pt-CaFe_PB_ in SAP

**Figure 8:**
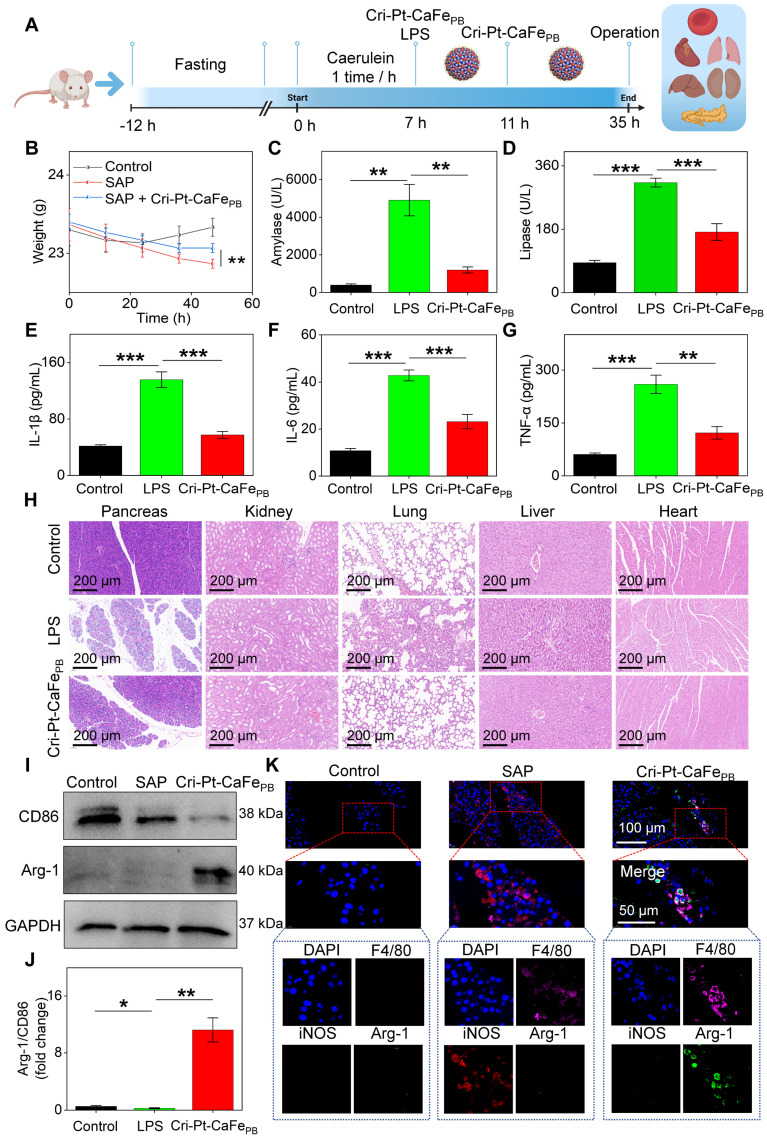
** Therapeutic effect of Cri-Pt-CaFe_PB_ in SAP mice. (A)** Schematic illustration of therapeutic protocol for Cri-Pt-CaFe_PB_ in SAP mice. **(B)** Changes in the weights of the mice with the time recorded within 47 h (n = 3). ELISA analysis of amylase **(C)**, lipase **(D)**, IL-1β **(E)**, IL-6 **(F)**, and TNF-α **(G)** in serum. **(H)** H&E staining of the pancreatic, kidney, lung, liver, and heart. **(I, J)** WB analysis of CD86 and Arg-1 protein levels in pancreatic tissue. **(K)** Immunofluorescence analysis of the number of macrophages (F4/80, pink fluorescence), iNOS (red fluorescence), and Arg-1(green fluorescence) in pancreatic tissues. ***P < 0.001, **P < 0.01, *P < 0.05.

Inspired by the remarkable ROS-scavenging activity of Cri-Pt-CaFe_PB_ and their ability to regulate cell polarization *in vitro*, we further investigated their therapeutic effect of Cri-Pt-CaFe_PB_ *in vivo* using an SAP mice model. As shown in Figure 8A, all mice were fasted for 12 h and then intraperitoneally injected with Caerulein and LPS to induce inflammation. Since the pathological features of the pancreas induced by Caerulein/LPS closely resemble those observed in humans 31, a mouse model of SAP has been successfully constructed after 7 h of reaction. Cri-Pt-CaFe_PB_ was subsequently administered via tail vein injection twice, at 7 and 11 h, respectively. After intervention for 35 h, blood samples and major organs, including heart, liver, pancreas, lung, and kidney, were collected to assess the anti-inflammatory therapeutic potential of Cri-Pt-CaFe_PB_ *in vivo*. Importantly, the weights of the mice recovered to varying degrees after intervention with Cri-Pt-CaFe_PB_ in SAP treatment, showing its good effect (Figure 8B). After coincubation with the nanozyme, the expression levels of inflammatory indicators such as serum amylase (Figure 8C), lipase (Figure 8D), IL-1β (Figure 8E), IL-6 (Figure 8F), and TNF-α (Figure 8G) are significantly reduced, indicating that Cri-Pt-CaFe_PB_ can alleviate SAP by regulating the inflammatory cytokine pathway *in vivo*. As shown in Figure 8H, H&E staining results reveal that the tissues of the mice in the SAP group present some typical inflammatory pathological changes, such as pancreatic cell edema, necrosis, local infiltration of neutrophils and lymphocytes, and bleeding spots. After two intravenous injections of Cri-Pt-CaFe_PB_, the pancreatic tissue of SAP mice recovered closely to normal tissue. Similar pathological improvements were observed in other organs, such as the kidneys, lungs, and liver (Figure 8H). But in contrast, other nanozymes, including CaFe-Pt_PB_, PtCa_PB_, and PtFe_PB_, did not show significant recovery in pancreatic tissue (Figure S12). Besides, we also summarize the therapeutic effects of other PB-based nanomaterials in various inflammatory diseases (Table S3). To further explore the anti-inflammatory mechanisms of Cri-Pt-CaFe_PB_ in SAP therapy, the changes in the expression levels of macrophages polarization-related proteins, such as CD86 (an M1 marker) and Arg-1 (an M2 marker), in different treatment groups were evaluated via western blotting technology. As shown in Figure 8I-J, the expression of CD86, associated with proinflammatory M1 macrophages, is downregulated, while the expression of Arg-1, associated with anti-inflammatory M2 macrophages, is restored. This result is in line with the expectations of SAP treatment. Moreover, the high level of inflammatory factors including serum amylase, lipase, IL-1β, IL-6, and TNF-α in SAP group indicates the successful construction of the SAP model (Figure 8B-J).

On the other hand, the immunofluorescence staining was also performed to locate M1 macrophages (iNOS and F4/80 markers) and M2 macrophages (Arg-1 and F4/80) in pancreatic tissues, whole-tissue scanning images were depicted in Figure S13. And Figure 8K shows a local view of the significant colocalization of iNOS and Arg-1 in pancreatic tissues subjected to different treatments. Compared to the SAP group, the number of M2/M1 macrophages significantly increased in the Cri-Pt-CaFe_PB_ group (Figure S14), confirming that the Cri-Pt-CaFe_PB_ nanozyme can effectively inhibited the polarization of M1 macrophages and promote the polarization of M2 macrophages *in vivo*. Collectively, these findings suggest a feasible strategy for SAP treatment by utilizing Cri-Pt-CaFe_PB_ as ROS scavenger to regulate macrophage polarization, shifting the balance from proinflammatory M1 to anti-inflammatory M2 macrophages.

Promoting macrophage polarization from M1 to M2 phenotype has been proven to possess therapeutic effects in a variety of inflammatory models, such as myocardial infarction inflammation 49, atherosclerosis 50, osteogenesis and bone repair 51. SAP is an inflammatory response involving multiple systems and organs, and the recruitment and activation of macrophages and their interactions with other cells promote the occurrence and progression of pancreatitis through a series of complex mechanisms. Therefore, researchers have explored the therapeutic mechanism of PB-based drugs for inflammatory diseases, including clearing ROS, inhibiting pathogenic endoplasmic reticulum stress, restoring defective autophagy, blocking c-Jun N-terminal kinase (JNK) phosphorylation, downregulating chronic inflammatory mediators, enhancing mitochondrial autophagy, and modulating macrophage polarization (Table S3). Notably, this study presented a cisplatin-based nanomedicine of Cri-Pt-CaFe_PB_, as a ROS scavenger, which not only inhibited the release of pro-inflammatory cytokines and mediators such as serum amylase, lipase, IL-6, IL-1β, and TNF-α, but also promoted the polarization of macrophage phenotype M1 to M2. DFT simulations also confirmed the therapeutic mechanism of Cri-Pt-CaFe_PB_ with high POD activity to the anti-inflammation/antioxidant effect. In addition, compared with other PB-based nanozymes (Table S2), Cri-Pt-CaFe_PB_ exhibited long-term stability, long-term biosafety, and good pharmacokinetics behavior* in vitro* and* in vivo*.

## Conclusions

In this study, we successfully engineered a cisplatin-like calcium hexacyanoferrate Prussian blue nanozyme (Cri-Pt-CaFe_PB_) by covalently coupling platinum (IV) with ultrasmall PEG-coated CaFe_PB_ nanoparticles. Importantly, Pt(IV) serves not only as a connector grafted onto the surface of CaFe_PB_, but also as a connector to the FAD-approved cisplatin (II) medicine for the formation of cisplatin- and Pt(IV)-conjugated nanozymes with high biosafety and multienzyme activities. This unique structure allows the synthesized Cri-Pt-CaFe_PB_ with robust antioxidant and anti-inflammatory capabilities that can protect cells from ROS-induced damage and maintain normal cellular metabolism. In addition, DFT simulations were also used to explore the meaningful therapeutic mechanism of Cri-Pt-CaFe_PB_ in detail, proving that its POD-like activity acts as a crucial role in its anti-inflammation behavior. Interestingly, the Cri-Pt-CaFe_PB_ can demonstrate its strong ability to differentiate proinflammatory M1 into anti-inflammatory M2 macrophages by clearing ROS in the SAP mouse model. And the Cri-Pt-CaFe_PB_ can further reduce the expression of proinflammatory cytokines, such as serum amylase, lipase, IL-6, IL-1β, and TNF-α *in vivo*, alleviating SAP symptoms. This discovery provides a feasible strategy and a new direction for employing cisplatin-like Prussian blue nanozymes as ROS scavengers and macrophages modulators in the effective cure of inflammatory/ROS-related diseases.

## Supplementary Material

Supplementary experimental section, figures and tables.
