# Supplementary material for: Modulating Macrophage Polarization for Severe Acute Pancreatitis Therapy via Cisplatin-like Prussian Blue Nanozymes
Source: Theranostics. 2025 Aug 11;15(17):8916–34. doi: 10.7150/thno.113523 (PMC12439267; doi:10.7150/thno.113523)
Supplement: Supplementary file 1 — Supplementary experimental section, figures and tables. [file thnov15p8916s1.pdf]

Supporting Information

Modulating macrophage polarization for severe acute pancreatitis therapy via cisplatin-like Prussian blue nanozymes

*Ling Wu[1, 2 †], Rui Cai[1, 2 †], Yuhang Li[2, 3, 4 †], Shuqi Liao[1, 2, 3], Yinghui Song[2, 3, 4], Yufeng Li[2, 3, 4], Jishan Li[5], Donghong Yu[6], Zhong Cao[1, *], Sulai Liu[2, 3, 4]**

[1] Hunan Provincial Key Laboratory of Materials Protection for Electric Power and Transportation & Hunan Provincial Key Laboratory of Cytochemistry, School of Chemistry and Pharmaceutical Engineering, Changsha University of Science and Technology, Changsha 410114, China

[2] Department of Hepatobiliary Surgery/Central Laboratory, Hunan Provincial People's Hospital (The First Affiliated Hospital of Hunan Normal University), Changsha 410005, China

[3] Hunan Engineering Research Center of Digital Hepatobiliary Medicine, Changsha 410005, China.

[4] Hunan Provincial Key Laboratory of Biliary Disease Prevention and Treatment, Changsha 410005, China.

[5] State Key Laboratory of Chemo/Bio-Sensing and Chemometrics, College of Chemistry and Chemical Engineering, Hunan University, Changsha 410082, China

[6] Department of Chemistry and Bioscience, Aalborg University, DK-9220 Aalborg, Denmark

----

[†] L. Wu, R. Cai, and Y. Li contributed equally to this work.

* Corresponding author. E-mail address: caoz@csust.edu.cn (Z. Cao); liusulai@hunnu.edu.cn (S. Liu)

Table of Contents

Experimental……S3

Materials……S3

Instrumentation……S4

DFT calculations……S4

Cell culture……S5

Cell toxicity assay……S5

Hemolysis in vitro……S5

Figures and Tables……S6

Figure S1 ……S6

Figure S2 ……S7

Figure S3 ……S8

Figure S4 ……S9

Figure S5 ……S10

Figure S6 ……S11

Figure S7 ……S12

Figure S8 ……S13

Figure S9 ……S17

Figure S10 ……S18

Figure S11 ……S19

Figure S12 ……S20

Figure S13 ……S23

Figure S14 ……S24

Table S1……S12

Table S2……S14

Table S3……S21

References……S25

Experimental

Materials

All reagents and chemicals were commercially available. Glacial acetic acid (CH_3COOH), sodium acetate (CH_3COONa), hydrochloric acid (HCl), sodium hydroxide (NaOH), absolute ethanol (CH_3CH_2OH), hydrogen peroxide (H_2O_2, 30%), ferrous sulfate heptahydrate ($FeSO_4 \cdot 7H_2O$), calcium chloride ($CaCl_2$), N, N-dimethylformamide (DMF), glutathione (GSH), and potassium hexacyanoferrate (III) ($K_3[Fe(CN)_6]$) were purchased from Sinopharm Chemical Reagent Co., Ltd. (Beijing, China). Tris(hydroxymethyl)aminomethane ($C_4H_{11}NO_3$), glutathione reductase (GR), 1,1-diphenyl-2-picrylhydrazyl (DPPH), reduced nicotinamide adenine dinucleotide phosphate (NADPH), polyetherimide (PEI), and indocyanine green (ICG) were ordered from Macklin Biochemical Co., Ltd. (Shanghai, China). 5,5-Dimethyl-1-pyrroline-N-oxide (DMPO) was obtained from Dojindo Laboratories (Kumamoto Ken, Japan). Polyvinylpyrrolidone (PVP, kW=30 K), hexahydrate chloroplatinic acid ($H_2PtCl_6 \cdot 6H_2O$), 3,3',5,5'-tetramethylbenzidine (TMB), Mal-PEG_{2000}-COOH (PEG), and pyrogallol (PG) were provided from Aladdin Biochemical Technology Co., Ltd. (Shanghai, China). DMEM, RPMI-1640 medium, paraformaldehyde fixative, lipopolysaccharide (LPS), physiological saline, and hematoxylin and eosin (HE) staining kits were bought from Servicebio Technology Co., Ltd. (Wuhan, China). 2',7'-Dichlorodihydrofluorescein diacetate (DCFH-DA) and 5-ethynyl-2'-deoxyuridine (EdU) were acquired from Beyotime Biotechnology (Shanghai, China). Horseradish peroxidase-conjugated goat anti-rabbit IgG was purchased from Proteintech (Chicago, USA). Anti-Arg-1 (1:1000) and anti-GAPDH (1:2000), and anti-CD86 (1:1000) were purchased from Affinity Biosciences (USA). The anti-iNOS (1:200), anti-Arg-1 (1:200), and anti-F4/80 (1:1000) were purchased from Cell Signaling Technology (Danvers, MA, USA). TNF-α, IL-1β, and IL-6 assay kits were obtained from Thermo Fisher Scientific (Waltham, MA, USA). Unless specified, all reagents used in this study were of analytical grade. All experimental water used was deionized water (resistivity $\Omega \geq 18.2$ MΩ cm). All animal experiments were performed at the First Affiliated Hospital of Hunan Normal University and were approved by the Institutional Animal Committee of Hunan Provincial People's.

Instrumentation

UV-vis absorption spectra were acquired via a UV-1900I ultraviolet-visible spectrophotometer (Shimadzu Corporation, Japan). X-ray photoelectron spectroscopy (XPS) analysis was conducted on an Escalab 250X-ray photoelectron spectroscope (Thermo Fisher Scientific Co. Ltd., USA). The crystallographic structure was achieved via X-ray powder diffraction (XRD, D/max-2500 with a source of Cu Kα radiation (λ=1.54056 Å), Rigaku Co., Ltd., Tokyo). Fourier transform infrared (FTIR) spectroscopy was performed with a Nicolet iS20 spectrometer (Thermo Fisher Scientific, USA). Transmission electron microscopy images were obtained via an FEI instrument (Tecnai G2 F20 TMP, USA) (TEM, JEM-F200) equipped with energy dispersive spectroscopy (EDS, JED-2300T) analysis. Dynamic light scattering (DLS) data were collected with a Brookhaven 90 plus analyzer (NanoBrook Omni, USA). Protein quantification was conducted via nucleic acid protein quantification analyzers (NanoDropone, Thermo Fisher Scientific, China). The staining state of the cells was observed via laser scanning confocal microscopy (Olympus FV3000, Olympus Corporation, Japan).

DFT calculations

The DFT computations were carried out using the projector augmented wave (PAW) method via the Vienna ab initio simulation package (VASP) [1, 2]. The exchange correlation potential was represented by the Perdew-Burke-Ernzerhof (PBE) function in the generalized gradient approximation (GGA) [3]. The total energy was corrected via the DFT-D3 method and used to describe van der Waals interactions [4]. A 2×2 supercell Cri-Pt-$CaFe_{PB}$ (111) surface was constructed. The vacuum layer was set to 15 Å. The cutoff energy for the plane-wave basis expansion was set to 450 eV, using a 2×2×1 *k*-point sampling grid. During the optimization process, the bottom five layers of atoms were fixed, while the other layers were relaxed. All the structures were optimized to have equivalent energy and convergence tolerance of force on the atoms below 1.0×10^{-5} eV and 4.0×10^{-2} eV/Å, respectively. The Gibbs free energy change (ΔG) of the elementary step was calculated as follows:

$$\Delta G = \Delta E + \Delta E_{ZPE} - T\Delta S \qquad (2)$$

whereas ΔE is the total energy difference, ΔE_{ZPE} is the change in the zero-point energy and the entropy, T is the temperature (T = 298.15 K), and ΔS is the entropy change.

Cell culture

RAW264.7 cells were cultured in Dulbecco's modified Eagle's medium (DMEM) (Gibco, China) supplemented with 10% fetal bovine serum (FBS) and 1% penicillin G sodium/streptomycin sulfate. AR42J cells were cultured in Dulbecco's modified Eagle's medium (DMEM) (Gibco, China) supplemented with 1% penicillin G sodium/streptomycin sulfate and 20% FBS (Gibco, China).

Cell toxicity assay

The cytotoxicity of Cri-Pt-$CaFe_{PB}$ was evaluated via the Cell Counting Kit-8 (CCK-8) assay. Briefly, AR42J cells s were seeded and incubated in a 96-well plate for 24 h. When the cell density was approximately 80%, the medium was replaced with fresh medium containing 100 μL of Cri-Pt-$CaFe_{PB}$ solution at various concentrations (0, 50, 100, 200, 400, 800, and 1600 μg/mL). After incubation at different time points (1, 6, 24, and 48 h), 10 μL of CCK-8 was added to each well, followed by incubation for 1 h at 37°C. The main active component of CCK-8, 2-(2-methoxy-4-nitrophenyl)-3-(4-nitrophenyl)-5-(2,4-disulfophenyl)-2H-tetrazolium monosodium salt (WST-8), is reduced in living cells by mitochondrial dehydrogenases to form a water-soluble orange formazan dye. The absorbance measured at 450 nm via a microplate reader can indirectly reflect the number of living cells. Each group was tested with five replicates (n=5).

Hemolysis in vitro

Blood samples (1.0 mL) were collected from healthy mice and centrifuged at 2000 rpm for 5 min to remove the supernatant. The red blood cells were washed three times with PBS, and a red blood cell suspension was prepared with 1 mL of PBS. Subsequently, 50 μL of the red blood cell suspension was added to a centrifuge tube along with 150 μL of various solutions (PBS, NaCl, H_2O, and Cri-Pt-$CaFe_{PB}$). After incubation for 4 h, the images were collected.

Figures and Tables

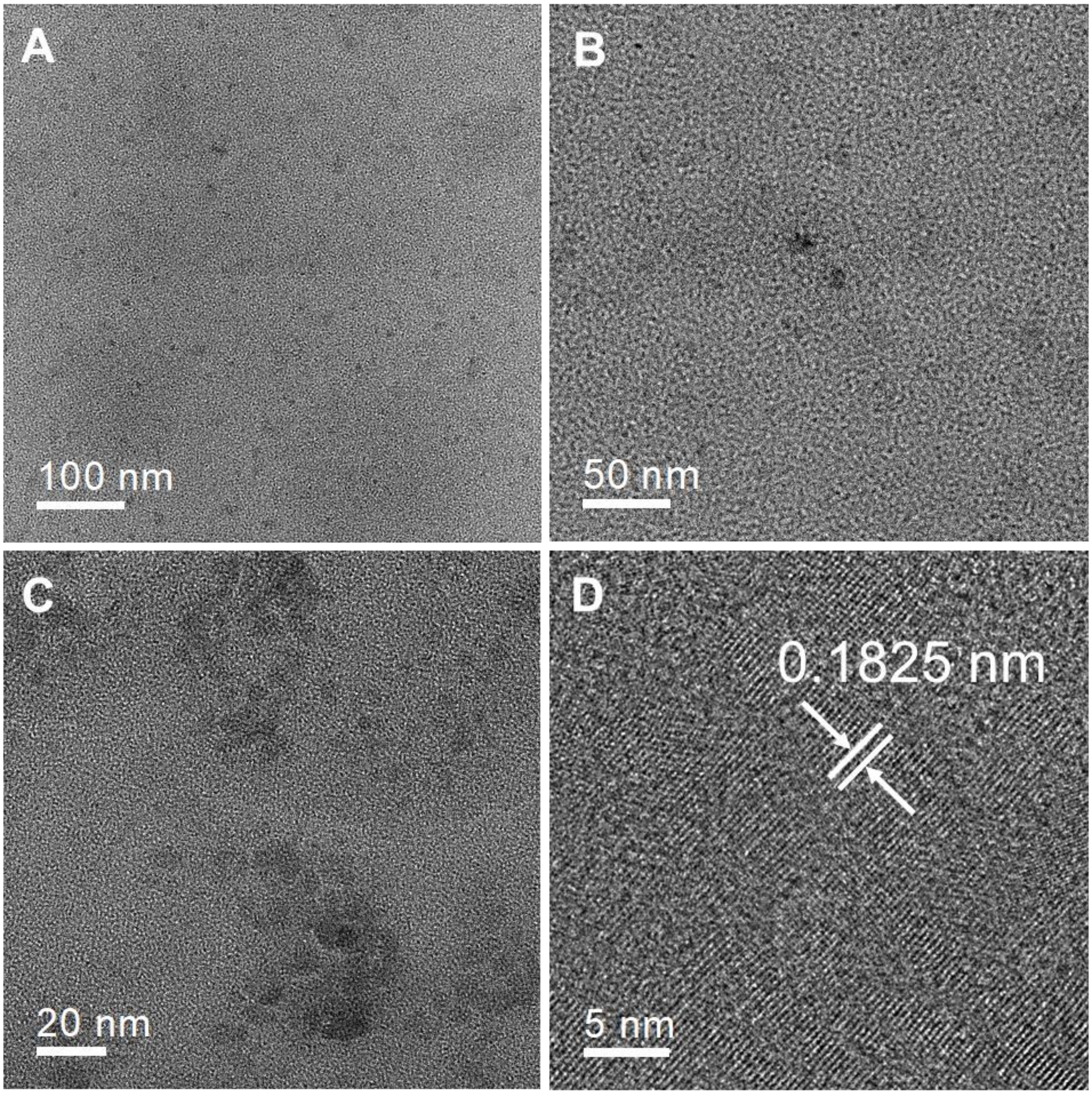

Figure S1. TEM images of $CaFe_{PB}$ nanomaterial at different magnifications with scale bar of (A) 100 nm, (B) 50 nm, and (C) 20 nm, respectively. (D) HR-TEM image of $CaFe_{PB}$ nanomaterial.

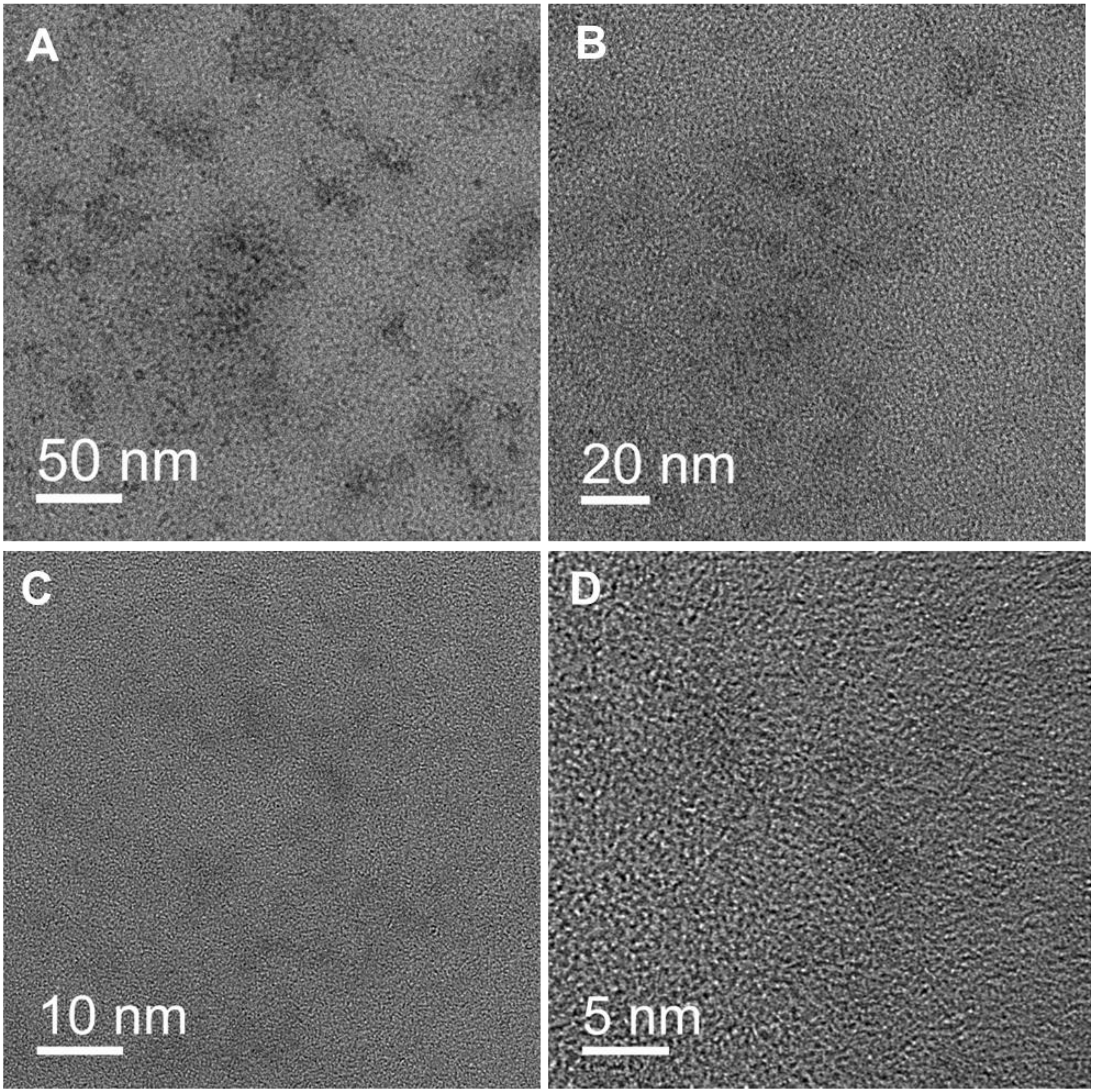

Figure S2. TEM images of $PtCa_{PB}$ at different magnifications with scale bar of (A) 50 nm, (B) 20 nm, (C) 10 nm, and (D) 5 nm, respectively.

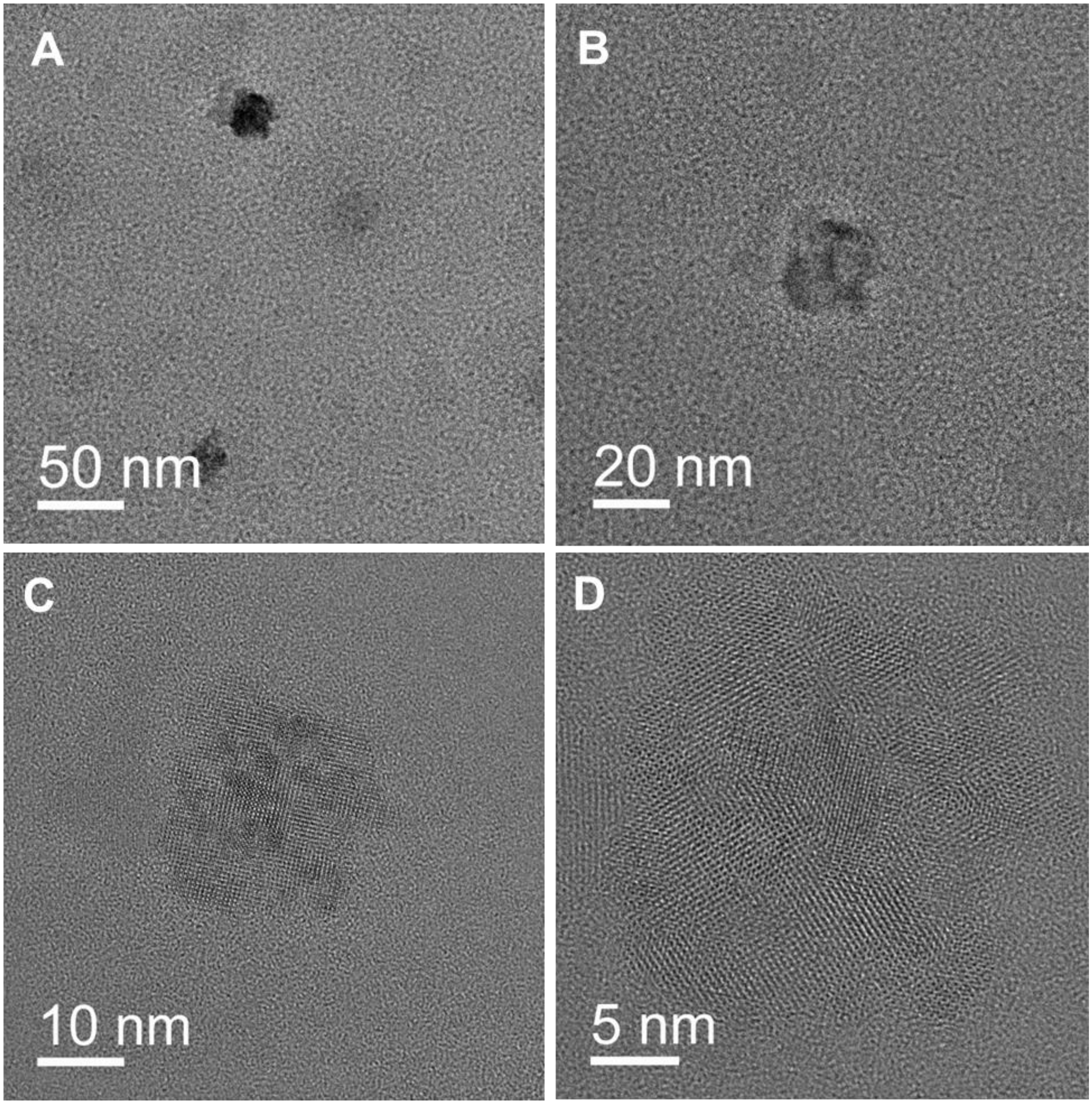

Figure S3. TEM images of $PtFe_{PB}$ at different magnifications with scale bar of (A) 50 nm, (B) 20 nm, (C) 10 nm, and (D) 5 nm, respectively.

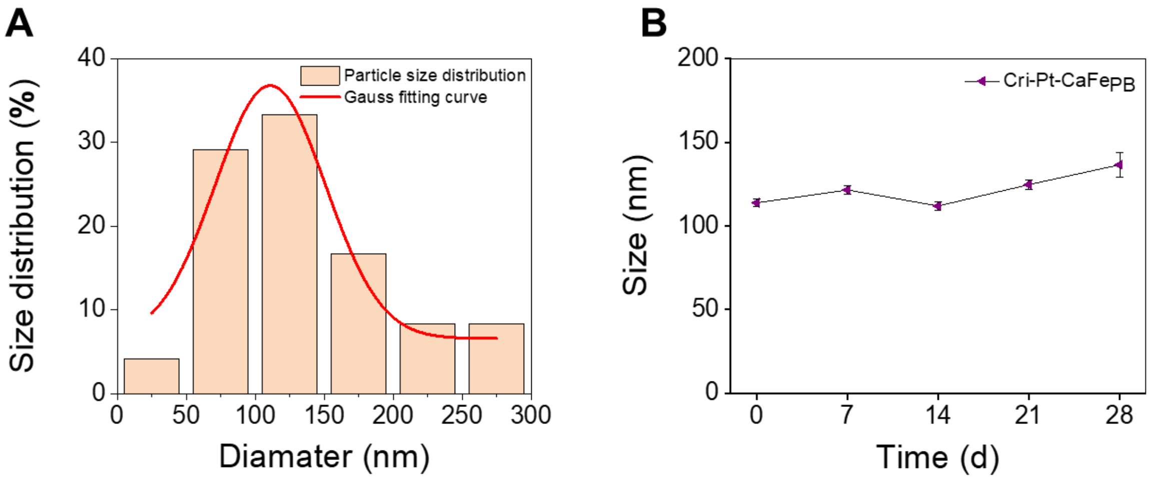

Figure S4. (A) The size distribution histogram of Cri-Pt-$CaFe_{PB}$ in an aqueous solution. (B) Long-term stability assessment of Cri-Pt-$CaFe_{PB}$ in an aqueous solution over 28 d (n = 3).

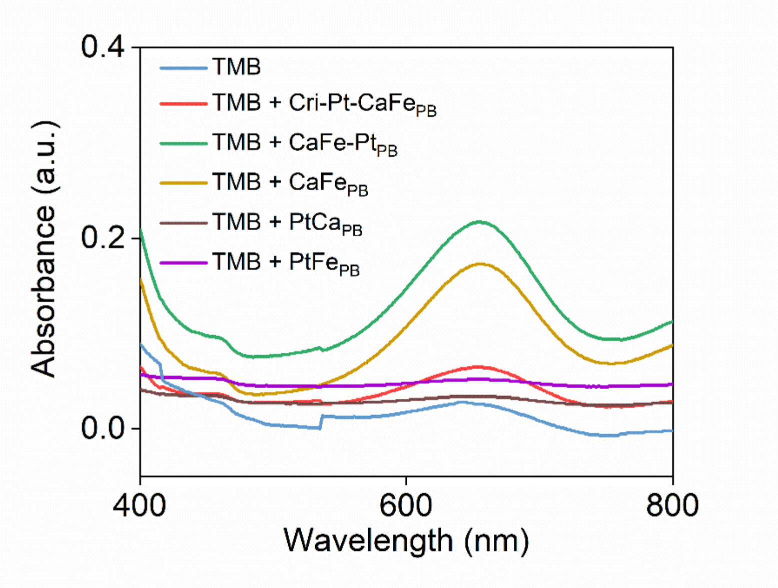

Figure S5. UV-vis absorption spectra of oxidase-like activity of different nanomaterials.

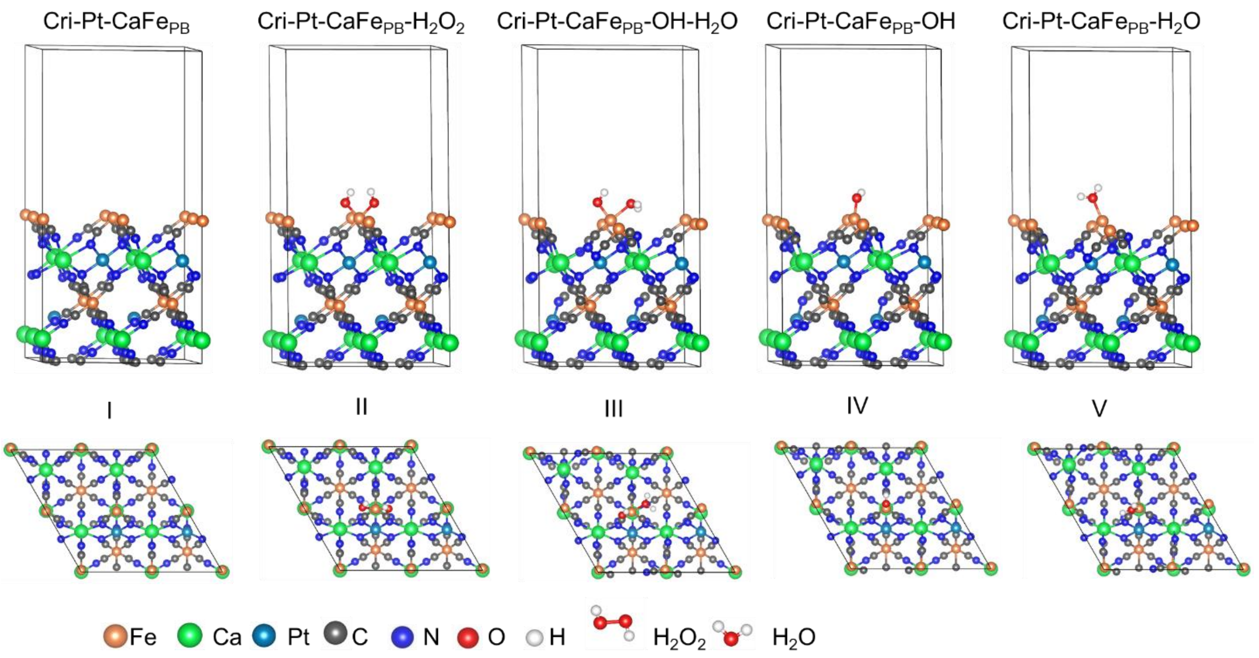

Figure S6. The optimized structure diagram of intermediate in reaction process: side view (top), top view (bottom). I: Cri-Pt-$CaFe_{PB}$, II: Cri-Pt-$CaFe_{PB}$-H_2O_2, III: Cri-Pt-$CaFe_{PB}$-OH-H_2O, IV: Cri-Pt-$CaFe_{PB}$-OH, V: Cri-Pt-$CaFe_{PB}$-H_2O.

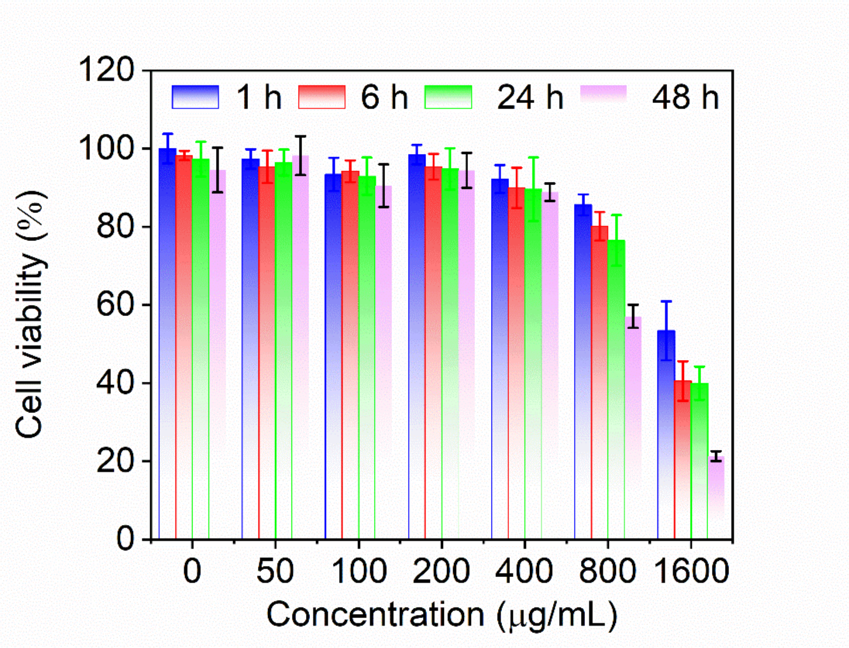

Figure S7. Cell vitality evaluation of Cri-Pt-CaFe$_{PB}$ with different concentrations on AR42J for 1, 6, 24, and 48 h by CCK-8 assay. The significant analysis between groups were depicted in Table S1.

Table S1. Statistical significance of cell vitality of AR42J Cells treated with different concentrations of Cri-Pt-CaFe$_{PB}$ at 1, 6, 24, and 48 h (CCK-8 Assay)

Time (h)	Dose (μg/mL)					
	50	100	200	400	800	1600
1	*ns*	*ns*	*ns*	*	***	***
6	*ns*	*ns*	*ns*	**	***	***
24	*ns*	*ns*	*ns*	*ns*	***	***
48	*ns*	*ns*	*ns*	*ns*	***	***

Notes: *ns*, not significant; *$p < 0.05$; **$p < 0.01$; ***$p < 0.001$ (compared with 0 μg/mL).

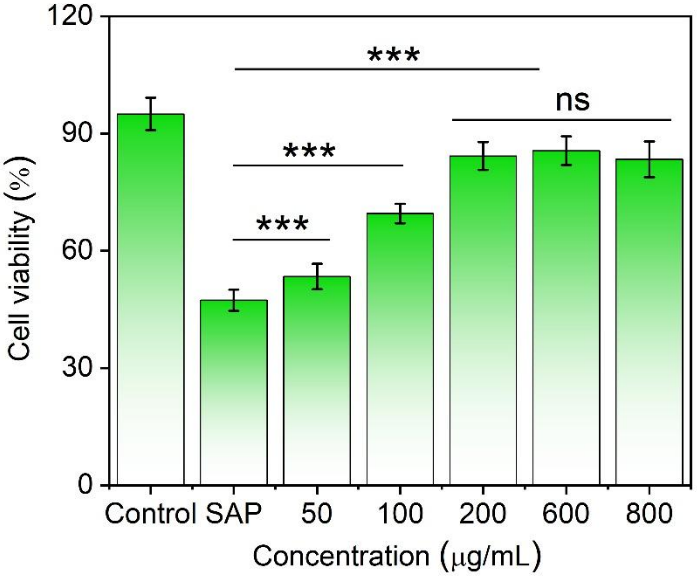

Figure S8. The protective effect of Cri-Pt-CaFe$_{PB}$ at different concentrations on LPS-induced RAW264.7 cells for 24 h. *$P \leq 0.05$, **$P \leq 0.01$, ***$P \leq 0.001$, and *ns* = not significant.

Table S2 *In vitro* and *in vivo* biocompatibility of PB-based nanozymes.

Prussian blue forms	Size (nm)	Biocompatibility	Disease	Ref.
PBzyme	~65 nm	In vitro: >90 % cell viable in HUVECs (Dosage: ~160 μg/mL) In vivo: -	Skin flaps	5
PBNPs	~160 nm	In vitro: >90 % cell viable in NPCs, 293T (Dosage: ~3.125 μg/mL) In vivo: Insignificant toxicity in SD rat (Dosage: 2 mg/mL, 28 days)	Intervertebral disc degeneration	6
PBzyme	~100 nm	In vitro: >90 % cell viable in BV2 (Dosage: ~100 μg/mL) In vivo: -	Ischemic stroke	7
PBzyme	~106 nm	In-vitro: >90 % cell viable inBV2, SH-SY5Y (Dosage: ~80 μg/mL) In vivo: Full metabolism in 30 days	Parkinson's disease	8
USPBNPs	~3.5 nm	In-vitro: >90 % cell viable in THP-1 (Dosage: ~10 μ g/mL) In vivo: -	Osteoarthritis	9
MPBzyme@NCM	~160 nm	In vitro: >90 % cell viable in BV2, bEnd.3, SH-SY5Y (Dosage: ~40 μg/mL) In vivo: -	Ischemic Stroke Therapy	10
PB@PDA NPs	~115 nm	In vitro: >90 % cell viable in mouse primary neuron, microglia, astrocytes (Dosage: ~80 μg/mL) In vivo: -	Adult ischemic stroke	11
HA-M@PB@(PC+ART)	~200 nm	In vitro: >90 % cell viable in RAW264.7, VSMCs, HUVECs (Dosage: ~100 μg/mL) In vivo: -	Atherosclerosis	12
Sim@PMPB NC	~150 nm	In vitro: >90 % cell viable in RAW264 (Dosage: ~400 μg/mL) In vivo: Insignificant toxicity in Kunming mice (Dosage:20 mg/kg, 28 days)	Atherosclerosis	13
PB/RBC	~74 nm	In vitro: >90 % cell viable in SH-SY5Y, bEnd.3, BV2	Alzheimer’s disease	14

		(Dosage: ~40 μg/mL)<br>In vivo: -		
RHPAzyme	~240 nm	In vitro: >90 % cell viable in SH-SY5Y<br>(Dosage: ~10 μg/mL)<br>In vivo: -	Spinal cord injury	15
PBNZs	4 nm	In vitro: >90 % cell viable in HEK239T<br>(Dosage: ~200 g/mL)<br>In vivo: Insignificant toxicity in BALB/C mice<br>(Dosage: 50 mg/kg, 14 days)	Acute kidney injury	16
KBP@KH	250 nm	In vitro: >90% cell viable in L929<br>(Dosage: ~100 μg/mL)<br>In vivo: -	Diabetic wound healing	17
PB@MSCM	150 nm	In vitro: >90% cell viable in BM Lin^-, MS-5<br>(Dosage: ~100 μg/mL)<br>In vivo: Insignificant toxicity in C57BL/6 mice<br>(Dosage: 15 mg/kg, 14 days)	Radiation-induced hematopoietic injury	18
PB Scavengers	~80 nm	In vitro: >90 % cell viable<br>(Dosage: ~100 μg/mL)<br>In vivo: Insignificant toxicity in C57BL/6JGpt mice<br>(Dosage: 1 mg/kg, 24 h)	Hepatic ischemia-reperfusion injury	19
PtPB	~110 nm	In vitro: murine mammary carcinoma (4T1) cells<br>(Dosage: ~160 ppm)<br>In vivo: hemolysis test<br>(Dosage: 200 μg/mL, 24 h)	Cancer therapy	20
Cri-Pt-$CaFe_{PB}$	~105 nm	In vitro: >93 % cell viable in AR42J.<br>(dosage: < 400 μg/mL)<br>In vivo: Insignificant toxicity in ICR mice<br>(Dosage: 5 mg/kg, 30 days)	SAP	This work

Note: HA-M@PB@(PC+ART): hyaluronic acid and macrophage membrane-coated Prussian blue nanoparticles; KBP@KH: bovine serum albumin Prussian blue- embedded Konjac glucomannan and hydroxypropyl trimethylammonium chloride chitosan composite hydrogel; MPBzyme@NCM: neutrophil-like cell-membrane-coated mesoporous Prussian blue nanozyme; PB: Prussian blue; PB@MSCM: mesenchymal stem cell membrane camouflaged Prussian blue nanozyme; PBNPs: Prussian blue nanoparticles; PBNZs: ultrasmall Prussian blue nanozymes; PB@PDA NPs: polydopamine-coated Prussian blue nanoparticles, PB/RBC: red blood cell membranes-encapsulated Prussian blue nanoparticles; PBzyme: Prussian blue nanozyme; PtPB: platinum-doped

Prussian blue; RHPAzyme: rapamycin-loaded and hollow mesoporous Prussian blue-based nanozyme; Sim@PMPB NC: simvastatin-loaded theranostic agent based on porous manganese-substituted Prussian blue analogues; USPBNPs: ultrasmall Prussian blue nanoparticles.

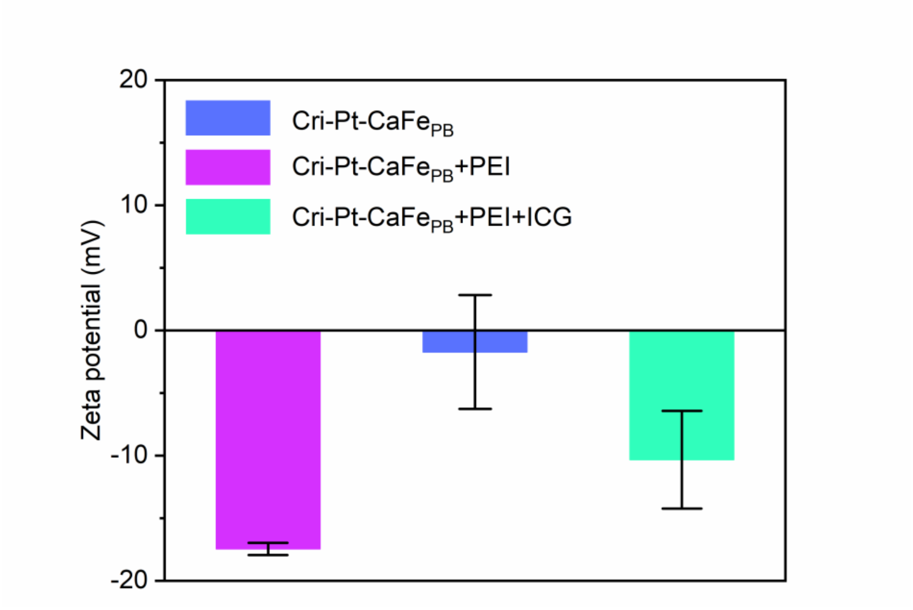

Figure S9. Zeta potential of various nanomaterials in an aqueous solution.

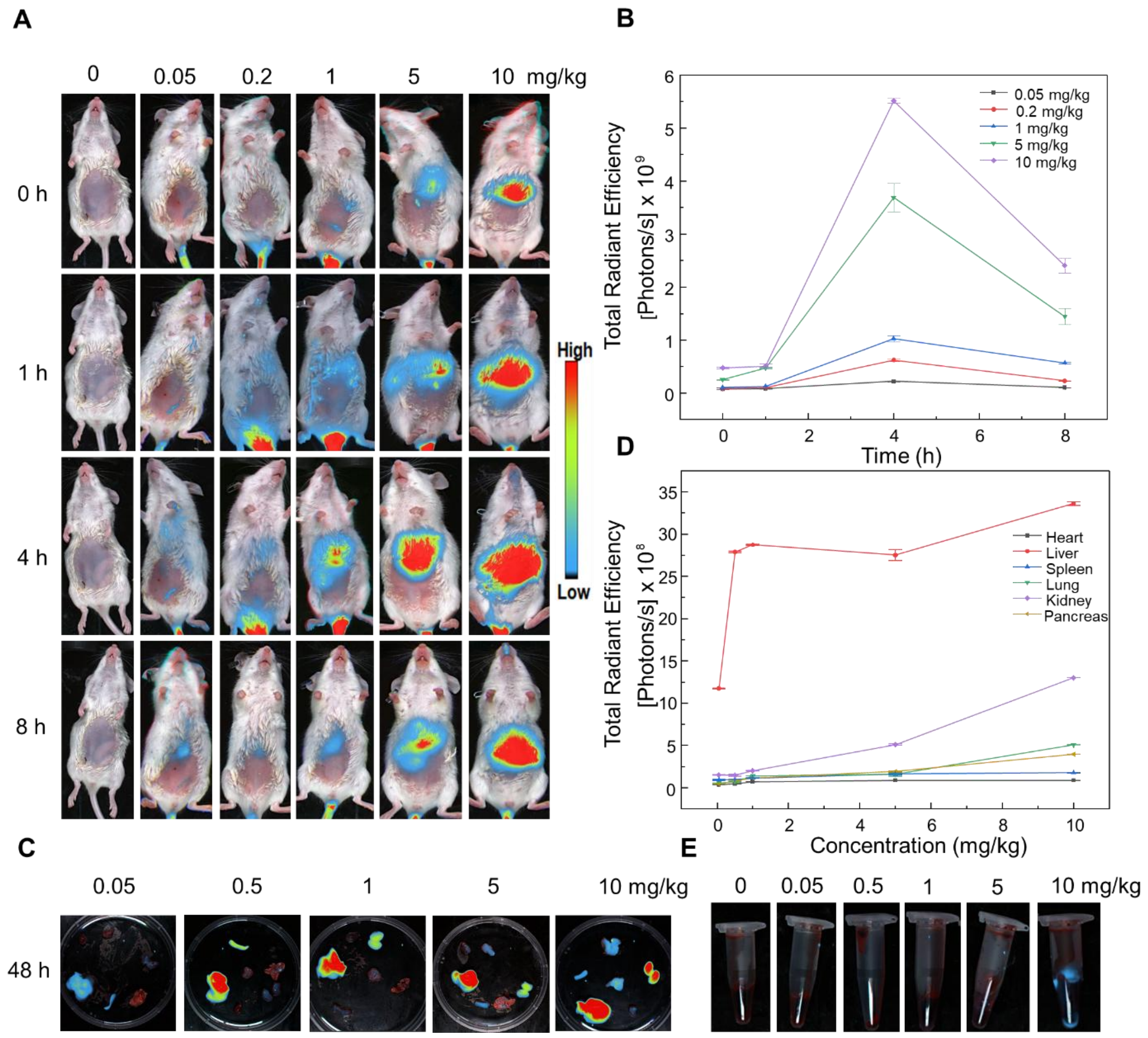

Figure S10. Fluorescence imaging (A) and corresponding fluorescence intensities (B) of ICG-labeled Cri-Pt-CaFe$_{PB}$ in mice with dose-dependent. Fluorescence images of dissected major organs (C) collected mice after injection of ICG-labeled Cri-Pt-CaFe$_{PB}$ with various concentration at 1, 4, 8 h, respectively. Fluorescence intensities (D) and corresponding fluorescence images (E) of ICG-labeled Cri-Pt-CaFe$_{PB}$ in blood at different dosage.

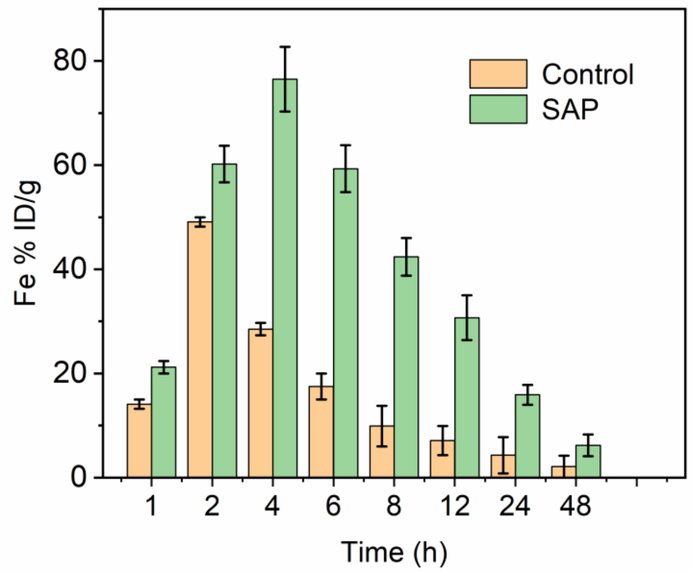

Figure S11. The changes in Fe elements of pancreatic tissue after injection of Cri-Pt-CaFe$_{PB}$ using ICP-MS at different time points (n = 3).

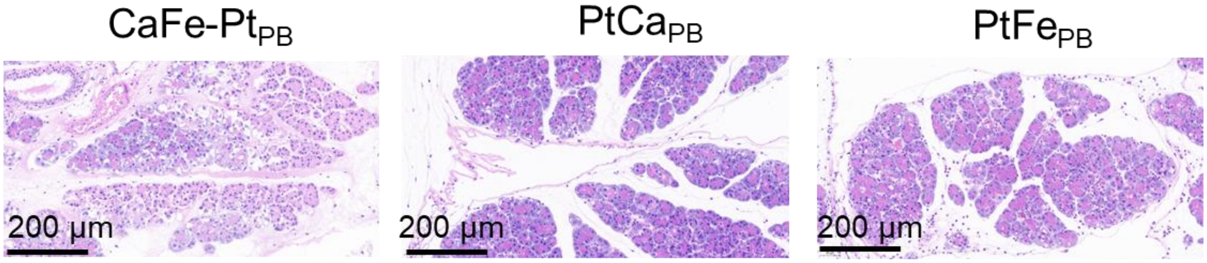

Figure S12. The H&E staining of the pancreas in SAP mice treated with CaFe-Pt_{PB}, $PtCa_{PB}$, and $PtFe_{PB}$ nanozymes.

Table S3 Current Prussian blue nanomaterials as enzyme mimics and their typical applications and representative references

Prussian blue forms	Enzyme-like activity	Mechanism	Application	Ref.
PBNPs@MCSs	CAT	ROS scavenging	Bone repair	21
MPBzyme@NCM	CAT	Inhibiting M1 responses	Ischemic stroke therapy	10
PBzyme	POD, SOD	Characteristic signal molecules of necroptosis	Ischemia-reperfusion(I/R) injury	5
PBNz@PSC	POD, SOD, CAT	Polarization from M1 toward M2	Myocardial Ischemia-Reperfusion Injury	22
USPBNPs	-	Polarization from M1 toward M2	Osteoarthritis	9
HMPB@BA#GaNPs	-	Inhibiting endoplasmic reticulum stress (IRE1/XBP1andATF4/CHOP axis) and restoring impaired autophagy (Beclin-1/p62/LC3axis)	Acute pancreatitis	23
PPBzymes	-	Blocking JNK phosphorylation	Osteoarthritis	24
SPBzyme	POD, SOD, CAT	Photothermal-enhanced NO release	Periodontal Disease	25
MPBZC	POD, SOD, CAT	ROS scavenging and enhancing mitochondrial autophagy	SAP	26
CPB-Ce6NPs	CAT	Upregulating VEGF	Antibacterial	27
KBP@KH	-	Inhibiting of chronic inflammatory factors	Diabetic wound healing	17
Cri-Pt-CaFe$_{PB}$	POD, SOD. GPx, RNS scavenging	RO/NS scavenging, inhibiting inflammatory responses, and inducing M1 to M2 macrophages repolarization	SAP	This work

Note: CPB-Ce6NPs: chlorin e6 loaded Prussian blue nanoparticles; HMPB@BA#Ga NPs: hollow mesoporous Prussian blue nanoparticles wrapped with neutrophil membranes and surface modified with the N,N-dimethyl-1,3-propanediamine moiety; KBP@KH: bovine serum albumin Prussian blue- embedded Konjac glucomannan and hydroxypropyl trimethylammonium chloride chitosan composite hydrogel; MPBZC: macrophage-membrane-coated and ZIF-8-modified PB-nanoparticles-loaded

celastrol; MPBzyme@NCM: neutrophil-like cell-membrane-coated mesoporous Prussian blue nanozyme; PB: Prussian blue; PBNPs@MCSs: Prussian blue nanohybridized multicellular spheroids; PBNz@PSC: Prussian blue nanozyme coated with polydextrose-sorbitol carboxymethyl ether; PPBzymes: Prussian blue nanozymes coated with Pluronic; SPBzyme: integrate sodium nitroprusside into PBzyme; USPBNPs: ultrasmall Prussian blue nanoparticles.

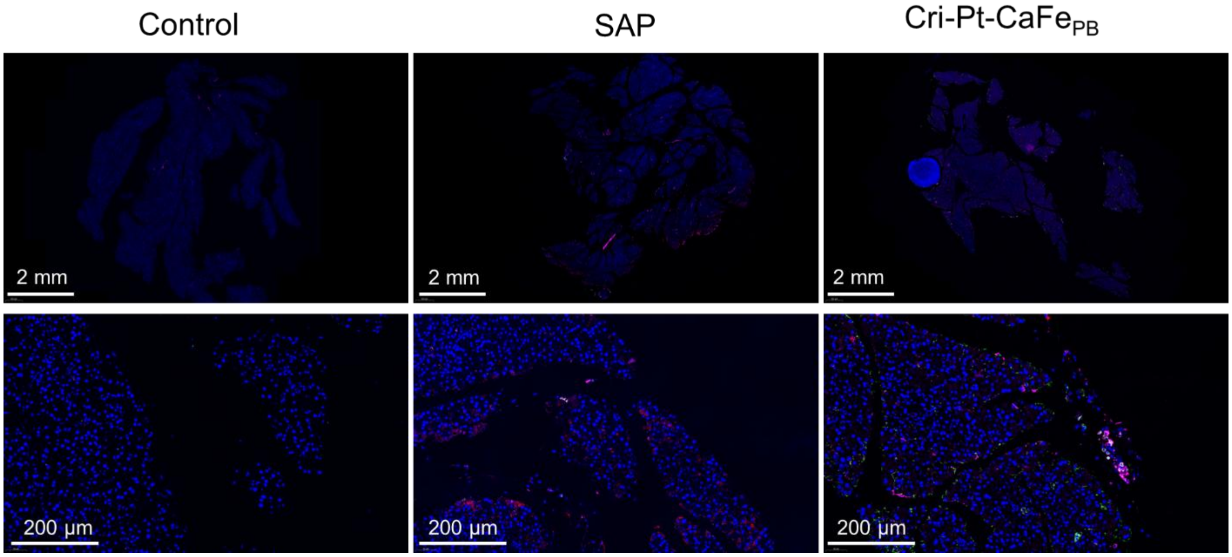

Figure S13. Whole-tissue scanning images of the number of macrophages (F4/80, pink fluorescence), iNOS (red fluorescence), and Arg-1(green fluorescence) in pancreatic tissues, scale bar 2 mm and 200 μm, respectively.

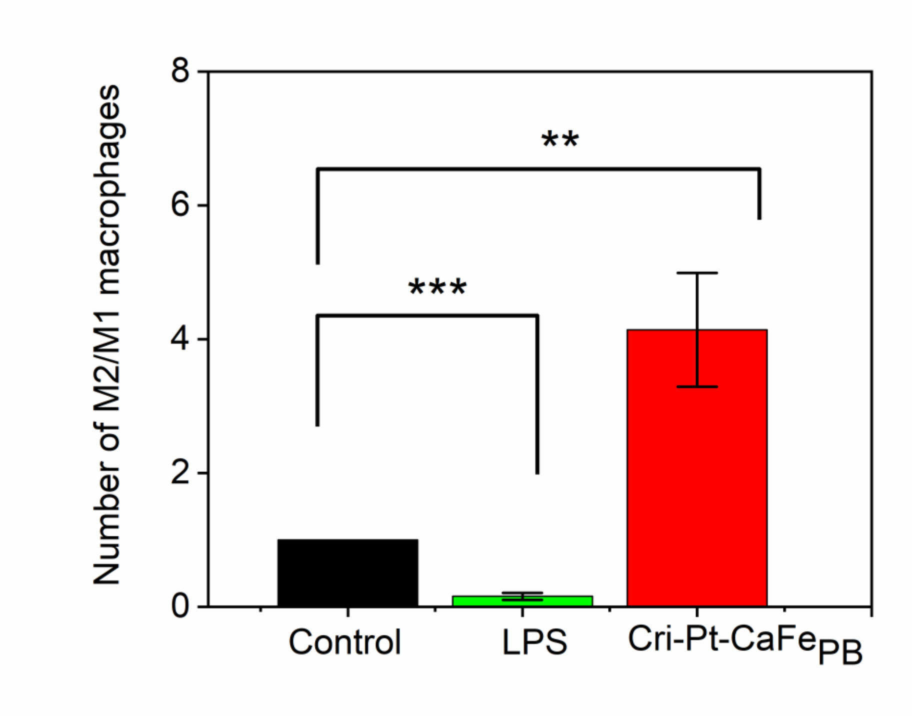

Figure S14. The quantitative colocalization ratios of the immunofluorescence images of M1/M2 macrophages.

3 References

1. Kresse G, Furthmüller J. Efficiency of ab-initio total energy calculations for metals and semiconductors using a plane-wave basis set. Comput Mater Sci. 1996; 6: 15-50.

2. Kresse G, Furthmüller J. Efficient iterative schemes for ab initio total-energy calculations using a plane-wave basis set. Phys Rev. 1996; 54: 11169-86.

3. Perdew JP, Burke K, Ernzerhof M. Generalized gradient approximation made simple. Phys Rev Lett. 1996; 77: 3865-8.

4. Oktavian R, Goeminne R, Glasby LT, Song P, Huynh R, Qazvini OT, et al. Gas adsorption and framework flexibility of CALF-20 explored via experiments and simulations. Nat Commun. 2024; 15: 3898.

5. Hou R, Lu T, Gao W, Shen J, Yu Z, Li D, et al. Prussian blue nanozyme promotes the survival rate of skin flaps by maintaining a normal microenvironment. ACS Nano. 2022; 16: 9559-71.

6. Zhou T, Yang X, Chen Z, Yang Y, Wang X, Cao X, et al. Prussian blue nanoparticles stabilize sod1 from ubiquitination-proteasome degradation to rescue intervertebral disc degeneration. Adv Sci. 2022; 9: e2105466.

7. Liu J, Sun J, Song Y, Wang M, Zhao P, Wang W, et al. Prussian blue nanozyme treatment of ischemic brain injury via reducing oxidative stress inhibits inflammation, suppresses apoptosis, and promotes neurological recovery. ACS Chem Neurosci. 2023; 14: 1535-46.

8. Ma X, Hao J, Wu J, Li Y, Cai X, Zheng Y. Prussian blue nanozyme as a pyroptosis inhibitor alleviates neurodegeneration. Adv Mater. 2022; 34: e2106723.

9. Qin Z, Li X, Wang P, Liu Q, Li Y, Gu A, et al. Ultrasmall prussian blue nanozyme attenuates osteoarthritis by scavenging reactive oxygen species and regulating macrophage phenotype. Nano Lett. 2024; 24: 11697-705.

10. Feng L, Dou C, Xia Y, Li B, Zhao M, Yu P, et al. Neutrophil-like cell-membrane-coated nanozyme therapy for ischemic brain damage and long-term neurological functional recovery. ACS Nano. 2021; 15: 2263-80.

11. Zhao Y, Song C, Wang H, Gai C, Li T, Cheng Y, et al. Polydopamine-cloaked nanoarchitectonics of prussian blue nanoparticles promote functional recovery in neonatal and adult ischemic stroke models. Biomater Res. 2024; 28: 0079.

12. Zhou H, You P, Liu H, Fan J, Tong C, Yang A, et al. Artemisinin and procyanidins loaded multifunctional nanocomplexes alleviate atherosclerosis via simultaneously modulating lipid

influx and cholesterol efflux. J Control Release. 2022; 341: 828-43.
13. Zhang Y, Yin Y, Zhang W, Li H, Wang T, Yin H, et al. Reactive oxygen species scavenging and inflammation mitigation enabled by biomimetic prussian blue analogues boycott atherosclerosis. J Nanobiotechnology. 2021; 19: 161.
14. Li L, Xiong Y, Zhang Y, Yan Y, Zhao R, Yang F, et al. Biofilm-camouflaged Prussian blue synergistic mitochondrial mass enhancement for Alzheimer's disease based on Cu^{2+} chelation and photothermal therapy. J Control Release. 2024; 375: 269-84.
15. Shen K, Li X, Huang G, Yuan Z, Xie B, Chen T, et al. High rapamycin-loaded hollow mesoporous Prussian blue nanozyme targets lesion area of spinal cord injury to recover locomotor function. Biomaterials. 2023; 303: 122358.
16. Zhang DY, Liu H, Zhu KS, He T, Younis MR, Yang C, et al. Prussian blue-based theranostics for ameliorating acute kidney injury. J Nanobiotechnology. 2021; 19: 266.
17. Tang DX, Liu K, Yang JY, Wang ZJ, Fu LL, Yang XJ, et al. Artificial nonenzymatic antioxidant Prussian blue/KGM-BSA nanocomposite hydrogel dressing as ROS scavenging for diabetic wound healing. Int J Biol Macromol. 2024; 266: 131106.
18. Zhang B, Chen G, Wu X, Li Y, Xiao Y, Li J, et al. Biomimetic Prussian blue nanozymes with enhanced bone marrow-targeting for treatment of radiation-induced hematopoietic injury. Biomaterials. 2023; 293: 121980.
19. Huang Y, Xu Q, Zhang J, Yin Y, Pan Y, Zheng Y, et al. Prussian blue scavenger ameliorates hepatic ischemia-reperfusion injury by inhibiting inflammation and reducing oxidative stress. Front Immunol. 2022; 13: 891351.
20. Li ZH, Chen Y, Sun Y, Zhang XZ. Platinum-doped prussian blue nanozymes for multiwavelength bioimaging guided photothermal therapy of tumor and anti-inflammation. ACS Nano. 2021; 15: 5189-200.
21. Chen L, Tang S, Zhang J, Zhong C, Xu X, Yan J, et al. Prussian blue nanohybridized multicellular spheroids as composite engraftment for antioxidant bone regeneration and photoacoustic tomography. ACS Nano. 2024; 18: 24770-83.
22. Long M, Wang L, Kang L, Liu D, Long T, Ding H, et al. Prussian blue nanozyme featuring enhanced superoxide dismutase-like activity for myocardial ischemia reperfusion injury treatment. ACS Nano. 2025; 19: 4561-81.

23. Wang Y, Wang X, Zhang X, Zhang B, Meng X, Qian D, et al. Inflammation and acinar cell dual-targeting nanomedicines for synergistic treatment of acute pancreatitis via Ca^{2+} homeostasis regulation and pancreas autodigestion inhibition. ACS Nano. 2024; 18: 11778-803.
24. Cho C, Oh H, Lee JS, Kang LJ, Oh EJ, Hwang Y, et al. Prussian blue nanozymes coated with Pluronic attenuate inflammatory osteoarthritis by blocking c-Jun N-terminal kinase phosphorylation. Biomaterials. 2023; 297: 122131.
25. Li Z, Fan X, Liu Y, Yue M, Wu T, Wang X, et al. Engineering mild-photothermal responsive and no donor prussian blue nanozymes using mild synthesis for inflammation regulation and bacterial eradication in periodontal disease. Adv Mater. 2025; 37: e2409840.
26. Wang L, Gao Z, Tian M, Liu L, Xie J, Chen M, et al. A nanosystem alleviates severe acute pancreatitis via reactive oxygen species scavenging and enhancing mitochondrial autophagy. Nano Lett. 2025; 25: 8644-54.
27. Tong A, Tong C, Fan J, Shen J, Yin C, Wu Z, et al. Prussian blue nano-enzyme-assisted photodynamic therapy effectively eradicates MRSA infection in diabetic mouse skin wounds. Biomater Sci. 2023; 11: 6342-56.
